# Symptom probability increases at low pollen exposure in adolescents with allergic rhinitis findings from the GINIplus and LISA birth cohorts

**DOI:** 10.1111/pai.70429

**Published:** 2026-07-15

**Authors:** Jonas Schmid, Patricia Grill, Claudia Flexeder, Elisabeth Thiering, Jeroen Buters, Inga Weßels, Carsten Schmidt‐Weber, Marie Standl, Viktoria Ocvirk

**Affiliations:** ^1^ Institute of Epidemiology Helmholtz Center Munich, German Research Center for Environmental Health Neuherberg Germany; ^2^ Institute for Medical Information Processing, Biometry and Epidemiology (IBE), Faculty of Medicine LMU Munich Germany; ^3^ Pettenkofer School of Public Health Munich Germany; ^4^ German Center for Child and Adolescent Health (DZKJ) Munich Germany; ^5^ Institute and Clinic for Occupational, Social and Environmental Medicine LMU University Hospital Munich Germany; ^6^ German Center for Lung Research (DZL) Giessen Germany; ^7^ Division of Metabolic and Nutritional Medicine, Department of Pediatrics, Dr. von Hauner Children's Hospital LMU University Hospital Munich Germany; ^8^ Center of Allergy & Environment (ZAUM), Member of the German Center for Lung Research (DZL) Technical University and Helmholtz Center Munich Munich Germany; ^9^ Center of Allergy & Environment (ZAUM) Technical University and Helmholtz Center Munich Munich Germany

**Keywords:** adolescents, allergic rhinitis, birth cohort, epidemiology, pollen, pollen allergy symptoms, seasonal allergic rhinitis

## Abstract

**Background:**

Pollen‐induced allergic rhinitis increases significantly with age among children and adolescents and is associated with reduced quality of life due to symptom severity, social impacts, and decreased school performance. This study examines associations between pollen exposure and allergic rhinitis symptoms in German adolescents.

**Methods:**

In the 15‐year follow‐up of the GINIplus and LISA birth cohorts at the Munich study area, monthly occurrence of nasal symptoms was assessed in 1594 participants. Pollen concentrations of birch, grass, ragweed, and mugwort between January 2010 and March 2014 were considered. Monthly individual pollen exposures (grains/m^3^) were assigned as the sum of all pollen types to which participants were sensitized. Associations between pollen exposure and monthly nasal symptoms were analyzed using adjusted generalized additive mixed‐effect models.

**Results:**

A non‐linear association between individual pollen exposure and nasal symptoms was observed, with symptom probability increasing significantly at low pollen concentrations and plateauing around 24 grains/m^3^ (birch) and 14 grains/m^3^ (grass). The non‐linear association was evident during birch pollen seasons as well as early and late grass pollen seasons. Symptom probability increased more strongly in participants sensitized to birch and grass compared to those sensitized to grass only during grass seasons.

**Conclusion:**

Even at low pollen concentrations, there is an increase in symptom probability of seasonal allergic rhinitis that remains elevated at high pollen concentrations in sensitized participants. Enhanced probability rates also remain in late seasons after the pollen peak. These findings show that allergic rhinitis patients cannot rely on high‐pollen warnings but require accurate measurements to avoid exposure and allergic symptoms.

**Key message**

Allergic rhinitis is the most common allergic disease, affecting more than 400 million people worldwide. The condition often leads to significant reductions in quality of life due to symptom severity, impacts on social interactions, and decreased school performance. In Germany, the lifetime prevalence of seasonal allergic rhinitis (hay fever) among children and adolescents was reported to be 11% in 2018, increasing with age from childhood into adolescence. In this study, a non‐linear association between individual pollen exposure and nasal symptoms in 15‐year‐old participants from the GINIplus and LISA birth cohorts in the Munich area was observed. This association was characterized by a significant increase at low pollen concentrations and a plateau onset at elevated concentrations. These findings highlight the importance of sensitive pollen monitoring to guide allergic rhinitis patients throughout the seasons. Informing patients that allergic symptoms can occur even at moderate or low pollen concentrations may support self‐management and improve quality of life. A significant association between pollen exposure and nasal symptoms can also be assumed in the second half of the pollen season. Associations in pollen exposure and nasal symptoms differed between sensitization profiles within grass pollen seasons. Enhanced probability rates remain in late seasons after the pollen peak. Symptom probability increased stronger in participants sensitized to birch and grass compared to those sensitized to grass only during grass seasons. These findings indicate that allergic symptoms may need to be addressed even during periods of low to moderate pollen concentrations. Further studies are needed to explore biological mechanisms underlying the observed differences and should investigate whether comparable results are obtained when analyzing daily data in pollen exposure.

## 1 INTRODUCTION

Allergic rhinitis is the most common allergic disease, affecting more than 400 million people worldwide.^1^ The condition often leads to significant reductions in quality of life due to symptom severity, impacts on social interactions, and decreased school performance.^2^, ^3^ In Germany, the lifetime prevalence of seasonal allergic rhinitis (hay fever) among children and adolescents was reported to be 11% in 2018,^3^ increasing with age from childhood into adolescence.^4^

The most prevalent allergic rhinitis symptoms are nasal complaints, including sneezing and an itchy nose, followed by ocular and pulmonary manifestations.^5^ Allergic rhinitis is commonly classified into perennial allergic rhinitis, primarily caused by indoor allergens, and seasonal allergic rhinitis, typically triggered by outdoor allergens.^2^ Evidence indicates that exposure to pollen triggers and exacerbates seasonal allergic rhinitis symptoms, leading to more frequent and severe nasal congestion, sneezing, itching, and teary eyes.^6^, ^7^ This association is more pronounced in adolescents compared to children, likely due to higher pollen sensitization rates.^6^

In recent years, the prevalence of allergic rhinitis^8^ and sensitization to pollen^9^ has increased in many countries. However, significant differences in prevalence rates persist both within and between countries, reflecting regional variations in environmental factors and allergen exposure.^10^, ^11^ In the context of pollen exposure in Germany, sensitization rates are highest for grass and birch pollen, followed by lower rates for ragweed, mugwort, alder, and hazel.^12^, ^13^

Although it is known that the exposure to pollen is associated with allergic rhinitis symptoms,^14^, ^15^ there is limited evidence about pollen abundance and reported symptoms of seasonal allergic rhinitis in adolescents in Germany on a population‐based level.^16^ Adolescence may be a critical period for allergic diseases because symptoms are common, persistent, and can strongly affect daily life during a phase of ongoing physical and emotional development.^17^ Moreover, there is a lack of research on how repeated or prolonged pollen exposure may alter the association between pollen concentrations and allergic rhinitis symptoms over time. Recent studies have indicated that prior exposure to pollen in one season may influence the severity of allergic symptoms in subsequent pollen seasons,^18^ and that responses to pollen may vary depending on whether individuals are sensitized to one or multiple pollen types.^19^ Exploration of potential adaptation processes^20^, ^21^ could provide valuable insights into temporal dynamics of allergic reactions.

The objective of this study is therefore to analyze the exposure‐response association between airborne pollen concentrations and nasal symptoms among 15‐year‐old adolescents from the Munich study center of the GINIplus and LISA birth cohorts. In‐depth analyses will investigate (1) associations within specific pollen seasons, (2) variations between early and late pollen seasons, and (3) differences between subgroups based on their sensitization status to pollen types from successive pollen seasons.

## 2 MATERIALS AND METHODS

### 2.1 Study design and setting

The German Infant Study on the Influence of Nutrition Intervention Plus Environmental and Genetic Influences on Allergy Development (GINIplus) and Influence of Lifestyle‐Related Factors on the Immune System and the Development of Allergies in Childhood (LISA) studies are two ongoing, prospective, population‐based German birth cohorts.^22^ In GINIplus, 5991 newborns were recruited between 1995 and 1998 in the two study centres, Munich and Wesel. The study consists of two arms: an interventional and an observational arm. The aim of the randomized, double‐blinded GINI intervention was to investigate the allergy‐preventive effects of three different hydrolyzed infant formulas compared to regular cow's milk‐based formula in children with a positive family history during the first four months of life. Children without a family history of allergic diseases, or whose parents did not want to participate in the intervention or lived outside the study region, were assigned to the non‐interventional, observational study arm. Both arms together represent a population‐based cohort. For LISA, 3097 newborns were recruited between 1997 and 1999 in the study centres, Munich, Leipzig, Bad Honnef, and Wesel. In both studies, healthy, mature newborns with a birth weight of more than 2500 g were included. Excluded were newborns whose mothers suffered from immunologically relevant chronic disease or from drug or alcohol abuse. Also, children whose parents were not sufficiently capable of the German language or lived more than 50 km from the study centre or planned to move away from the study region were not included in the study. Details of the study design and recruitment procedures for the GINIplus and LISA birth cohorts have been previously described.^22^ Data for the analysis were obtained from the 15‐year follow‐up questionnaires of both birth cohorts, conducted between January 2010 and March 2014, with follow‐up rates of 53.4% (GINIplus) and 56.2% (LISA). At the 15‐year follow‐up, both studies were harmonized using identical study protocols, data collection procedures, and biosample collection methods as well as questionnaires. Therefore, both studies were combined for the subsequent analyses, adjusted for study cohort and study group (GINIplus intervention vs. GINIplus observation vs. LISA).^23^ Both studies were approved by local ethics committees, and written consent was obtained from the participants and their legal guardians. Only participants that took part in sensitization tests, provided answers to questions regarding symptoms, and resided in the Munich study area, for which the pollen data was available, were included in this analysis (Figure S1).

### 2.2 Data sources and variables

#### 2.2.1 Definition of the outcome variable

Parent‐administered questionnaires were used to collect information on nasal symptoms, defined as the primary outcome for allergic rhinitis in this analysis. Symptoms were assessed using questions from the International Study of Asthma and Allergies in Childhood (ISAAC) Phase One questionnaire manual.^24^ Parents were first asked whether their child had experienced “sneezing or a runny, blocked, or itchy nose in the past 12 months without having a cold”. If answered affirmatively, they were asked to specify the months within the past year when their child experienced nasal symptoms, allowing multiple selections across all 12 months. For each month, a binary variable was generated to indicate the presence or absence of nasal symptoms based on the responses reported, resulting in 12 person‐months with symptom data per participant.

#### 2.2.2 Allergen sensitization

Allergen‐specific serum immunoglobulin E (IgE) concentrations from blood samples were assayed using the CAP‐RAST FEIA system (Pharmacia Diagnostics, Freiburg, Germany) according to the manufacturer's instructions, as described previously.^25^ The overall screening test SX1 was used to detect sensitization to inhalant allergens. If the SX1 test was positive (IgE >0.35 kilo units per liter [kU/L]), single allergen tests including birch, timothy grass, ragweed, mugwort, cat dander, and house dust mites (Dermatophagoides pteronyssinus) were performed. Participants were classified as sensitized to a specific allergen if IgE antibodies exceeded 0.35 kU/L.^26^

#### 2.2.3 Covariates

Age at questionnaire completion, sex, parental education (defined as the highest school grade completed by either parent, classified as low/medium for ≤10 years of education versus high (>10 years of education)), family atopy (any parent ever having asthma, eczema, or hay fever), passive smoking at home in the past 12 months, ever being diagnosed with asthma or eczema, time spent outside (number of hours spent outside on a working day during summer and winter, grouped into: 0–2 h, 2–5 h, >5 h) and sensitization to house dust mites or cat dander to account for perennial allergic rhinitis were selected for adjustment in the analysis. Medication use (treatment of hay fever symptoms in the previous 12 months) was excluded as a covariate from the analysis to avoid potential collider bias but was considered in sensitivity analyses. Missing data proportion for any adjustment variable was less than 5%. Participants with missing information were assigned to an “unknown” category.

#### 2.2.4 Pollen data

Pollen data was available for the Munich study center only. A Hirst‐type volumetric pollen trap, installed at the Clinic and Polyclinic for Dermatology and Allergology of the University of Munich, measured daily ambient pollen concentrations in grains/m^3^.^27^ Numbers of birch, grass, mugwort, and ragweed pollen, which have been identified among pollen types with the highest sensitization rates in Germany,^12^, ^13^ were included in the analysis.

#### 2.2.5 Pollen exposure

For each of the four pollen types, daily pollen concentrations in grains/m^3^ were aggregated into monthly mean pollen concentrations. To account for the participants' sensitization status, an individual pollen exposure (IPE) index was determined for each participant and month, as proposed by Luyten et al.,^5^ by summing up the monthly mean concentrations of only those pollen types to which the respective participant is sensitized. By restricting the exposure metric to sensitized pollen types, the IPE reflects individual immunological relevance and may therefore differ between participants with different sensitization profiles, despite shared ambient pollen conditions. This approach aims to better approximate the total pollen load experienced by each participant over time and provides a way to account for months that lie fully or only partly within the pollen seasons.

### 2.3 Statistical analysis

During the winter months, the pollen trap was deactivated due to an absence of pollen and concentrations were set to zero. For pollen seasons from March to October, missing daily pollen concentrations were imputed using the moving mean interpolation technique^28^ within the R‐package AeRobiology.^29^ Generalized additive mixed effect models (GAMM) with a logit link function^30^ were applied to analyze the exposure‐response association between IPE and symptom occurrence. Random intercepts for each participant were included to account for repeated measurements. The models were adjusted for age, sex, family atopy, passive smoking, allergic comorbidities, time spent outside, study cohort and sensitization to house dust mites or cat dander. All statistical analyses were performed using R software version 4.3.1.^31^ The GAMM models were constructed using the bam function from the mgcv R package.^32^ Smooth terms for mean IPE were modeled with cubic regression splines. Season‐specific analyses were performed for birch and grass pollen seasons, for which the onset of pollen seasons could be ascertained. Early versus late season analyses were conducted only for grass pollen, as sufficient season length of multiple months was required to define distinct phases. Early seasons were defined as the period from season onset up to the season peak and late season as the second period after the peak until the end of the season. An interaction term for season phase was included to analyze differences in symptom responses between early and late phases, by specifying season‐specific smooth terms within the GAMM framework. In a separate analysis, sensitization status was included as an interaction term by distinguishing participants sensitized only to grass (monosensitized) from those sensitized to both birch and grass (polysensitized), reflecting the predominant aeroallergens with consistently available exposure data. Smooth terms from the GAMM models were extracted to visualize the association between IPE and symptom occurrence and to assess whether exposure–response relationships differed across subgroups. The predict function from the mgcv package was employed to provide predictions of symptom occurrence by accounting for all predictors. Predictors were held constant at their mean values for continuous variables and at their most frequent categories for categorical variables. Several sensitivity analyses were conducted to assess the robustness of findings, including stratified analyses by study (GINIplus/LISA), sex, season of questionnaire completion, and medication use. The analyses were also repeated with a restricted population of participants who did not change their address from birth to 15 years. Further details are in Appendix S1.

## 3 RESULTS

### 3.1 Study participants

*N* = 1474 participants were included in the analysis with complete data on symptom reports and sensitization status, resulting in a total of 17,688 person‐months (Figure S1). Participants experiencing symptoms were more likely to have a family history of atopy and comorbidities of asthma or eczema, were treated for hay fever more often during the last 12 months, and were more frequently enrolled in the GINI intervention group (Table 1).

**TABLE 1:** Characteristics of study participants with at least one month of nasal symptom occurrence and participants without symptom occurrence, stratified by study (GINIplus/LISA).

	GINIplus			LISA		
	Overall	Symptoms^a^	No symptoms^a^	Overall	Symptoms^a^	No symptoms^a^
Overall	975	350	625	499	160	340
Sex						
Female	489 (50.2%)	168 (48.0%)	321 (51.4%)	239 (47.9%)	75 (46.9%)	164 (48.4%)
Age						
Mean (SD) in Years	14.6 (0.3)	14.5 (0.3)	14.6 (0.3)	14.5 (0.2)	14.5 (0.2)	14.5 (0.2)
Family atopy^b^						
Atopy mother	250 (25.6%)	92 (26.3%)	158 (25.3%)	198 (39.7%)	53 (33.1%)	79 (23.3%)
Atopy father	211 (21.6%)	69 (19.7%)	142 (22.7%)	132 (26.5%)	33 (20.6%)	65 (19.2%)
Atopy mother and father	246 (25.2%)	123 (35.1%)	123 (19.7%)	98 (19.6%)	31 (19.4%)	40 (11.8%)
Passive smoking^c^						
Yes	82 (8.4%)	35 (10.0%)	47 (7.5%)	34 (6.8%)	9 (5.6%)	25 (7.4%)
Unknown	16 (1.6%)	5 (1.4%)	11 (1.8%)	4 (0.8%)	1 (0.6%)	3 (0.9%)
Comorbidity^d^						
Asthma	58 (5.9%)	38 (10.9%)	20 (3.2%)	35 (7.0%)	23 (14.4%)	12 (3.5%)
Eczema	168 (17.2%)	72 (20.6%)	96 (15.4%)	74 (14.8%)	28 (17.5%)	46 (13.6%)
Asthma and Eczema	48 (4.9%)	33 (9.4%)	15 (2.4%)	14 (2.8%)	11 (6.9%)	3 (0.9%)
Unknown	21 (2.2%)	9 (2.6%)	12 (1.9%)	4 (0.8%)	0 (0.0%)	4 (1.2%)
Medication use^e^						
Medication used	136 (13.9%)	125 (35.7%)	11 (1.8%)	67 (13.4%)	64 (40.0%)	3 (0.9%)
Unknown	22 (2.3%)	13 (3.7%)	9 (1.4%)	4 (0.8%)	2 (1.3%)	2 (0.6%)
Time spent outside						
0–2 h /daily	318 (32.6%)	108 (30.9%)	210 (33.6%)	148 (29.7%)	48 (30.0%)	100 (29.5%)
2–5 h/daily	546 (56.0%)	198 (56.6%)	348 (55.7%)	304 (60.9%)	96 (60.0%)	208 (61.4%)
>5 h/daily	86 (8.8%)	36 (10.3%)	50 (8.0%)	33 (6.6%)	12 (7.5%)	21 (6.2%)
Unknown	25 (2.6%)	8 (2.3%)	17 (2.7%)	14 (2.8%)	4 (2.5%)	10 (2.9%)
Parental education						
Low/medium	203 (20.8%)	78 (22.3%)	125 (20.0%)	90 (18.1%)	37 (23.3%)	53 (15.7%)
Study arm						
GINI intervention	545 (55.9%)	224 (64.0%)	321 (51.4%)	‐	‐	‐
Pollen sensitization						
Grass	367 (37.6%)	231 (66.0%)	136 (21.8%)	159 (31.9%)	103 (64.4%)	56 (16.5%)
Birch	279 (28.6%)	190 (54.3%)	89 (14.2%)	105 (21.0%)	74 (46.3%)	31 (9.1%)
Mugwort	188 (19.3%)	135 (38.6%)	53 (8.5%)	73 (14.6%)	53 (33.1%)	20 (5.9%)
Ragweed	193 (19.8%)	134 (38.3%)	59 (9.4%)	79 (15.8%)	55 (34.4%)	24 (7.1%)
Indoor allergen sensitization						
House dust mites	281 (28.8%)	162 (46.3%)	119 (19.0%)	136 (27.3%)	74 (46.3%)	62 (18.3%)
Cat dander	175 (17.9%)	116 (33.1%)	59 (9.4%)	66 (13.2%)	44 (27.5%)	22 (6.5%)
Mean Individual pollen exposure^f^						
Mean (SD) in grains/m^c^	4.9 (7.7)	9.0 (8.5)	2.6 (6.1)	7.6 (13.7)	16.5 (16.2)	3.5 (9.9)
Median (Min–Max) in grains/m^c^	0.0 (0.0–361.67)	10.9 (0.0–222.53)	0.0 (0.0–361.67)	0.0 (0.0–361.67)	5.8 (0.0–361.67)	0.0 (0.0–361.67)

^a^ Symptom group includes participants with ≥1 month of nasal symptom occurrence during the study period.

^b^ Family member atopic and predisposed to asthma, eczema or hay fever.

^c^ Smoking in participants home in the last 12 months.

^d^ Asthma and/or eczema in the participants lifetime.

^e^ Hay fever treatment in the last 12 months.

^f^ Mean monthly individual pollen exposure based on sensitization to included pollen type.

### 3.2 Pollen exposure

**FIGURE 1:**
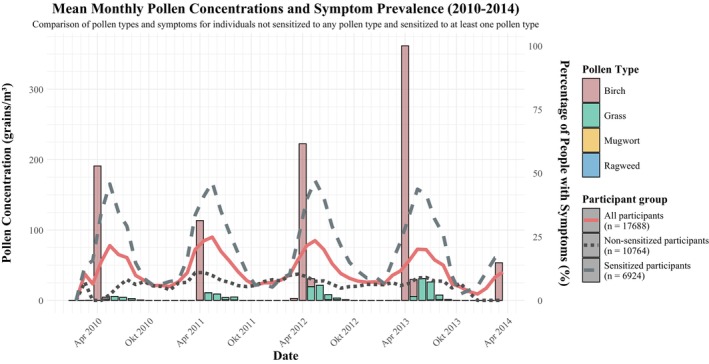
Mean monthly pollen concentrations for birch, grass, mugwort, and ragweed in grains/m^3^ (left y‐axis) and symptom prevalence (right y‐axis) of nasal symptoms in all participants (*n* = 17,688 person‐months) and groups of sensitized (*n* = 6924 person‐months) and non‐sensitized participants (*n* = 10,764 person‐months) from January 2010 to March 2014 (sensitized participants are sensitized to at least one of the four pollen types).

Birch and grass pollen concentrations were predominant throughout the study period (Figure 1). The birch pollen seasons started in late March to early April and lasted until late April to mid‐May, with the highest levels in April 2013. Grass pollen seasons started in early May to late May and ended in late June to early September, with the strongest peak observed in June 2013. Due to low pollen concentrations, no season onset could be determined for mugwort and ragweed pollen. Ragweed and mugwort were therefore not further considered in season‐specific analyses.

### 3.3 Symptom frequency

A total of 2200 months (12.4%) with nasal symptoms were reported. Symptom prevalence rates peaked in May, ranging from 20.1% in 2013 to 24.9% in 2011 (Figure 1). Among participants sensitized to at least one pollen type, prevalence rates peaked in May 2012 at 51.2%. Highest prevalence rates in participants not sensitized to any of the four pollen types were recorded in April 2011 at 11%. The prevalence rates of symptoms followed a seasonal trend in the group sensitized to pollen, with peaks recorded during pollen seasons. In contrast, no clear seasonal differences were detected in non‐sensitized participants (Figure 1).

### 3.4 Individual pollen exposure and probability of symptoms

**FIGURE 2:**
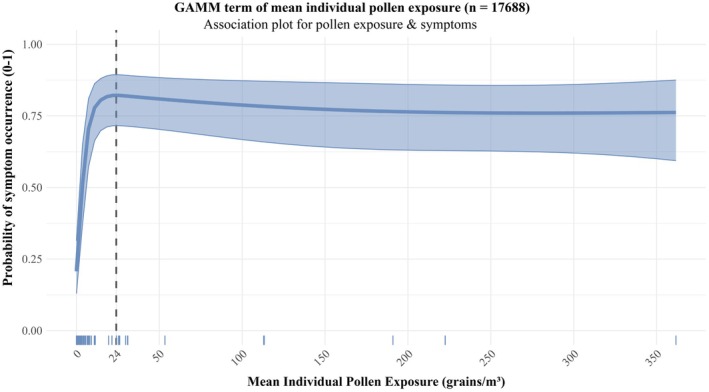
GAMM curves of the partial association of mean individual pollen exposure on probability of nasal symptom occurrence in the total study period. The solid line represents the model estimate, and the shaded areas represent the 95% confidence intervals. The horizontal line at x = 24 indicates the plateau value. Colored rug lines along the axes illustrate the distribution of data points and n represents the number of person‐months included in the model. Models were adjusted for age, sex, family atopy, passive smoking, asthma or eczema, time spent outside, and sensitization to house dust mites or cat dander.

The GAMM model of the overall association between IPE and symptom occurrence revealed a significant non‐linear association, with increasing symptom probability immediately at exposure onset (Figure 2). The GAMM model's smooth term for IPE did not show a minimum pollen exposure level necessary to initiate this increase (Figure 2). The predicted probability of symptoms plateaued at 47% (95% CI: 31%–63%) at pollen concentrations of approximately 24 grains/m^3^ (Figure S2). The results of the analyses stratified by sex (Figure S5), cohort (Figure S7), and date of questionnaire completion (Figure S8), as well as analyses including the covariate medication use (Figure S9), were consistent with those of the main analysis. For participants living at the same address from birth to 15 years, predicted symptoms similarly plateaued at approximately 24 grains/m^3^, with a probability of 38% (95% CI: 18%–64%) (Figure S10). Sensitivity analysis results are further described in Appendix S1. The predictors of allergic rhinitis symptom occurrence included in the model are illustrated in a forest plot (Figure S6).

#### 3.4.1 Associations in birch and grass pollen seasons

##### Birch pollen season

**FIGURE 3:**
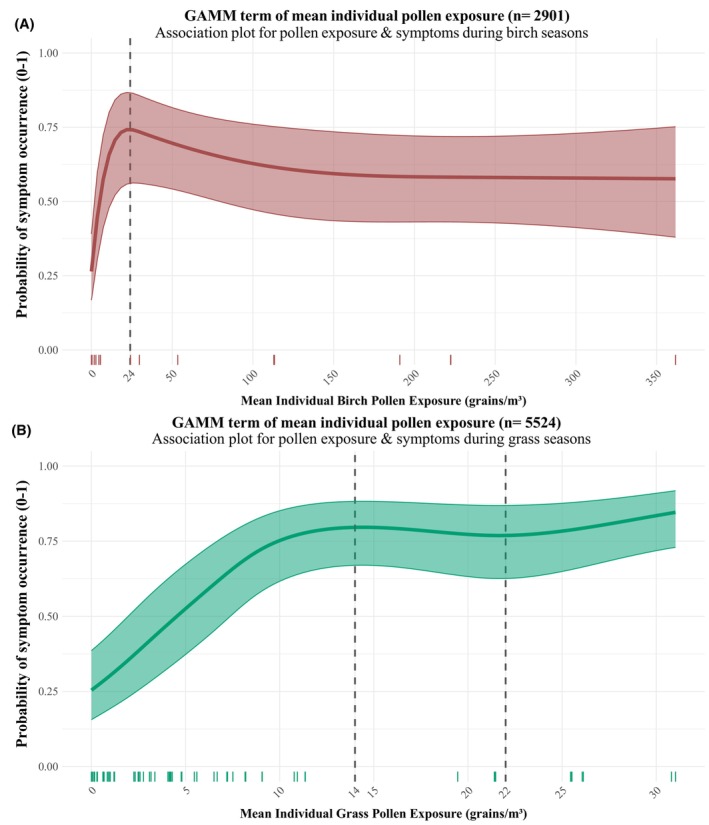
GAMM curves of the partial association of mean individual pollen exposure on probability of nasal symptom occurrence during (A) birch seasons and (B) grass seasons. The solid lines represent the model estimates, and the shaded areas represent the 95% confidence intervals. The horizontal lines indicate plateau values. Colored rug lines along the x‐axis illustrate the distribution of data points, and n represents the number of person‐months included in the analysis. The span of the x‐axis is adapted to the maximum of the respective pollen type. Models were adjusted for age, sex, family atopy, passive smoking, asthma or eczema, time spent outside, and sensitization to house dust mites or cat dander.

The association between IPE and symptom occurrence within birch pollen seasons (Figure 3A) revealed a comparable pattern to the results for the entire study period (Figure 2). Symptom probability increased immediately at exposure onset, followed by a plateau at a predicted probability of 56% (95% CI: 36%–74%) (Figure 3A) after approximately 24 grains/m^3^ (Figure 3A). Beyond this concentration, the GAMM curve indicated no significant increase in symptom probability with higher pollen levels (Figure 3A).

##### Grass pollen season

A continuous increase in the probability of nasal symptoms after pollen onset was subsequently followed by a first plateau at a predicted probability of 58% (95% CI: 44%–71%) (Figure S3B), at approximately 14 grains/m^3^ (Figure 3B). A second increase in the GAMM term beyond 22 grains/m^3^ was not statistically significant (Figure 3B).

##### Early versus late grass pollen season

**FIGURE 4:**
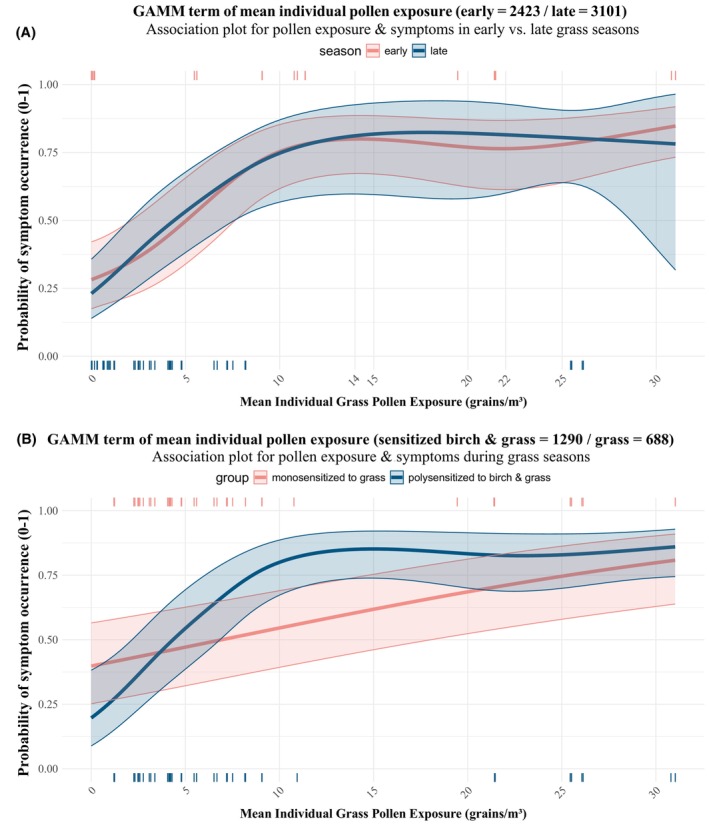
GAMM curves of the partial association of mean individual pollen exposure on probability of nasal symptom occurrence during (A) early vs. late grass seasons and (B) grass seasons in participants sensitized to grass and birch and grass but not birch. The solid lines represent the model estimates, and the shaded areas represent the 95% confidence intervals. Colored rug lines along the x‐axis illustrate the distribution of data points, and n represents the number of person‐months included in the analysis. Models were adjusted for age, sex, family atopy, passive smoking, asthma or eczema, time spent outside, and sensitization to house dust mites or cat dander.

In stratified analyses of the early and late grass pollen seasons, the probability of symptoms increased significantly with onset of pollen exposure in both periods, with no statistically significant difference between them (Figure 4A). The subsequent flattening of the GAMM curves showed no differences and occurred at similar pollen concentrations (Figure 4A) with predicted symptom probabilities of 58% (95% CI: 44%–71%) in early and 60% (95% CI: 36%–83%) in late seasons at 14 grains/m^3^ (Figure S4A).

##### Monosensitized Versus polysensitized participants during grass seasons

To investigate whether birch pollen sensitization alters the probability of symptom occurrence during grass pollen seasons, participants sensitized to both pollen types (polysensitized) were compared with those sensitized to grass pollen but not to birch pollen (monosensitized). Polysensitized participants showed a steep increase in symptom probability with rising grass pollen concentrations, followed by a plateau at elevated pollen levels (Figure 4B) with a predicted probability of 68% (95% CI: 54%–81%) at 14 grains/m^3^. In contrast, no plateau was observed in monosensitized participants, and a linear association was identified between symptom probability and grass pollen concentrations with a slower increase of symptom probability at lower pollen concentrations (Figure 4B) and a predicted probability of 36% (95% CI: 23%–48%) at 14 grains/m^3^ (Figure S4B).

## 4 DISCUSSION

This study investigated the association between pollen exposure and allergic rhinitis symptoms in 15‐year‐old participants of the GINIplus and LISA birth cohorts in the Munich area. A non‐linear association was found during birch pollen seasons as well as early and late grass pollen seasons. Associations in pollen exposure and nasal symptoms differed in grass pollen seasons between participants sensitized to birch and grass pollen and participants sensitized to grass pollen only.

### 4.1 Increased symptom prevalence in sensitized participants

Increasing prevalence rates of nasal symptoms were observed in months with detectable pollen concentrations in participants sensitized to at least one analyzed pollen type. An increase in symptom prevalence could be explained by allergic immune reactions to inhaled pollen. These reactions are possibly triggered by eosinophilic inflammation, with pollen exposure promoting eosinophil persistence through reduced apoptosis and thereby sustaining airway inflammation^33^ and are symptomatically classified as seasonal allergic rhinitis.^14^ A notable proportion of asymptomatic adolescents were sensitized to grass and birch, reflecting common “silent” sensitization where IgE positivity does not translate into clinical symptoms, as also reported by Eder et al., who showed that sensitization frequently occurs in the absence of clinical allergy.^34^

### 4.2 Non‐linear association between individual pollen exposure and nasal symptoms

Our study demonstrated a non‐linear association between IPE and nasal symptoms. The association is characterized by a steep increase in symptoms at low pollen concentrations, followed by a flattening of the exposure‐response curve. These findings underscore the need for targeted interventions on allergic rhinitis symptoms starting already at pollen exposure onset with lower pollen concentrations. Research that has previously investigated the association between pollen exposure and nasal symptoms primarily focused on specific pollen types rather than on a general overview including several pollen types. Only one study evaluated overall IPE and nasal symptoms, focusing on severity rather than occurrence. A non‐linear pattern with an increase at pollen onset followed by a plateau, consistent with our findings, was reported.^5^

#### 4.2.1 Increase in symptom occurrence at low pollen concentrations in birch and grass seasons

The analysis of pollen exposure and nasal symptoms within specific pollen seasons also showed a non‐linear association in the birch and grass pollen seasons. There were no indications of a necessary minimum pollen concentration for either birch or grass pollen until an increase in symptom probability was observed. Although some studies have observed pollen threshold levels below which no symptoms occured,^32^ our results are in line with previous findings from studies conducted in France and Australia.^35^, ^36^ Both studies reported no evidence of a minimum pollen concentration threshold below which symptoms didn't occur for birch or grass pollen. While low pollen levels were associated with symptoms, ragweed and mugwort were excluded from season‐specific analyses because their concentrations were consistently very low and any potential effects would likely have been masked by the substantially higher and more dominant pollen loads of birch and grass. Overall, these findings suggest that allergic immune reactions may emerge in sensitized individuals even at minimal pollen concentrations of birch and grass pollen.

#### 4.2.2 Plateau onset in symptom occurrence in birch and grass seasons

The estimated average daily pollen exposure at which nasal symptoms plateaued in this analysis was approximately 24 grains/m^3^ for birch and 14 grains/m^3^ for grass pollen. Several studies have already shown the appearance of a plateau when modeling the association between pollen exposure and nasal symptoms.^17^, ^35^, ^36^, ^37^, ^38^ In birch pollen, the plateau emerged at pollen concentrations ranging from 30 grains/m^3^ in Australia^37^ to 110 grains/m^3^ in France.^35^ Concerning grass pollen and the occurrence of nasal symptoms, the association curve attenuated at pollen concentrations of 30 grains/m^3^ in Spain^38^ and 80 grains/m^3^ in Germany^17^ and France.^36^ Comparisons with plateau pollen concentrations obtained in other studies must be made with caution. Differences in symptom outcomes, duration of pollen exposure,^39^ statistical methods, pollen allergenicity,^40^, ^41^ and study population across regions^34^ may explain variations in threshold numbers between countries.

Not all identified studies found a non‐linear association, with both linear^42^ and inverse associations^16^ reported between pollen exposure and allergic rhinitis symptoms. Nevertheless, our study consolidates the results of most studies demonstrating that symptom occurrence stabilizes above a certain pollen concentration yet remains at a consistently high level. While this underscores the considerable impact on symptoms at elevated pollen concentrations, repeated assessment of pollen levels and allergic responses could help health authorities refine measures and allocate resources when concentrations are predicted to exceed plateau levels.

#### 4.2.3 No difference in the associations between early and late pollen seasons

There were no significant differences in the association between pollen exposure and nasal symptoms between early and late grass pollen seasons, consistent with the results of the majority of related studies.^17^, ^43^, ^44^ However, it has been shown that symptoms of allergic rhinitis are more pronounced in the early season at similar grass pollen concentrations than in the late season.^45^ This may be due to increased inflammation and allergenic potency of pollen in the early season, combined with reduced perceived symptoms in the later season.^45^, ^46^ Research suggests that repeated exposure to pollen may reduce pollen concentrations required to induce allergic symptoms (priming effect),^20^ or that repeated exposure may lead to a down‐regulation of allergic responses.^21^ However, priming and adaptation mechanisms remain unclear in recent literature, and evidence on how these processes interact across consecutive pollen seasons is still limited.^17^, ^43^, ^44^ The findings of our analysis, in concordance with the results of most studies^17^, ^43^, ^44^ have not been able to confirm these mechanisms. Consequently, a substantial increase in symptoms of allergic rhinitis in adolescents may be expected in the presence of pollen concentrations in the late phase of pollen seasons too.

#### 4.2.4 Non‐linear association in polysensitized versus linear association in monosensitized participants

Participants sensitized to birch and grass pollen exhibited a non‐linear exposure‐response curve in the grass seasons. Participants who were sensitized to grass pollen alone showed a linear association. There are few studies on adaptation processes over subsequent pollen seasons. A possible priming effect of birch pollen on grass pollen has not yet been demonstrated.^45^ However, polysensitized individuals appear to have stronger allergic sensitization than monosensitized individuals.^47^ This is associated with an increased likelihood of rhinoconjunctivitis^19^ and lower allergen thresholds,^48^ which might explain the steep increase at low pollen concentrations. In addition, possible cross‐reactivities between birch and grass pollen^47^, ^49^ could explain differences between the groups.

### 4.3 Strength and limitations

The study's primary strength lies in the large number of participants, drawn from population‐based cohorts, with a long follow‐up period and exhaustive data on allergic symptoms and potential confounders. This is further complemented by the assessment of sensitization for targeted exposure attribution. The inclusion of several pollen types and data from multiple years enabled a robust analysis of the association between individual pollen exposure and nasal symptoms.

However, several limitations have to be considered when interpreting our findings. Although both studies are population‐based, they suffer from non‐random loss to follow‐up as every longitudinal cohort study. Participants included in the analysis are of higher socioeconomic status, which might limit the generalizability of our findings. One limitation is that parent‐reported symptom data may be prone to recall bias, as parents may not fully observe or accurately remember their adolescents' monthly symptoms, leading to possible misclassification of both timing and presence of symptoms. In addition, medication use may be prone to recall bias, and the assessment of symptom occurrence alone does not capture symptom severity, which may have led to an incomplete representation of the true symptom burden. Furthermore, misclassification of symptoms due to conditions other than allergic rhinitis cannot be excluded. Recruitment within the 15‐year follow‐up period does not allow to rule out attrition bias and could influence the generalizability of the results. Data on symptoms was also only collected monthly, which does not allow more detailed analyses with daily resolution to evaluate a potential lag effect or impact of overlapping pollen seasons.^5^, ^50^ However, non‐linear associations were also identifiable at the monthly level. The limited number of symptom data points without detailed information on their severity may have resulted in uncertainties, particularly at elevated concentrations or precise estimates of threshold values. Additionally, polysensitization was defined as sensitization to grass and birch pollen only. Thus, our findings cannot be directly compared with studies defining polysensitization as three or more allergen sensitizations, nor can we assess whether highly polysensitized adolescents exhibit increased symptom probability at low pollen concentrations. Furthermore, using data from a single pollen monitoring station cannot fully capture location‐dependent variability in exposure.^51^ Nevertheless, findings from Munich, where two stations 4 km apart showed only limited differences,^25^ support the view that one station can represent pollen levels within a surrounding area of about 30 km^25^. Possible environmental factors such as air pollution,^37^ weather conditions^50^ or prevailing pollen types^5^ were not considered in the analysis and could have contributed to symptom occurrence.

In this study, a non‐linear association between individual pollen exposure and nasal symptoms was observed, characterized by a significant increase at low pollen concentrations and a plateau onset at elevated concentrations. These findings highlight the importance of sensitive pollen monitoring to guide allergic rhinitis patients throughout the seasons. Informing patients that symptoms may occur even at low or moderate pollen concentrations could promote self‐management and improve quality of life. A significant association between pollen exposure and nasal symptoms can also be assumed in the second half of the pollen season. Associations in pollen exposure and nasal symptoms differed between sensitization profiles within grass pollen seasons. Further studies are needed to explore biological mechanisms underlying the observed differences and should investigate whether the results from this analysis also exist when analyzing daily data in pollen exposure.

## AUTHOR CONTRIBUTIONS

**Carsten Schmidt‐Weber:** Writing – review and editing. **Jonas Schmid:** Conceptualization; methodology; formal analysis; writing – original draft. **Inga Weßels:** Writing – review and editing. **Jeroen Buters:** Writing – review and editing. **Marie Standl:** Conceptualization; methodology; formal analysis; writing – review and editing. **Patricia Grill:** Writing – review and editing; formal analysis. **Viktoria Ocvirk:** Conceptualization; methodology; formal analysis; writing – review and editing. **Claudia Flexeder:** Methodology; formal analysis. **Elisabeth Thiering:** Formal analysis.

## FUNDING INFORMATION

GINIplus: The GINIplus study was mainly supported for the first 3 years of the Federal Ministry for Education, Science, Research, and Technology (interventional arm) and Helmholtz Zentrum Munich (former GSF) (observational arm). The 4, 6, 10, and 15 year follow‐up examinations of the GINIplus study were covered from the respective budgets of the 5 study centers (Helmholtz Zentrum Munich (former GSF), Research Institute at Marien‐Hospital Wesel, LMU Munich, TU Munich and from 6 years onwards also from IUF—Leibniz Research‐Institute for Environmental Medicine at the University of Düsseldorf) and a grant from the Federal Ministry for Environment (IUF Düsseldorf, FKZ 20462296). Further, the 15‐year follow‐up examination of the GINIplus study was supported by the Commission of the European Communities, the 7th Framework Program: MeDALL project, and as well by the companies Mead Johnson and Nestlé. LISA: The LISA study was mainly supported by grants from the Federal Ministry for Education, Science, Research, and Technology and in addition from Helmholtz Zentrum Munich (former GSF), Helmholtz Centre for Environmental Research—UFZ, Leipzig, Research Institute at Marien‐Hospital Wesel, Pediatric Practice, Bad Honnef for the first 2 years. The 4 year, 6 year, 10 year, and 15 year follow‐up examinations of the LISA study were covered from the respective budgets of the involved partners (Helmholtz Zentrum Munich (former GSF), Helmholtz Centre for Environmental Research—UFZ, Leipzig, Research Institute at Marien‐Hospital Wesel, Pediatric Practice, Bad Honnef, IUF—Leibniz‐Research Institute for Environmental Medicine at the University of Düsseldorf) and in addition by a grant from the Federal Ministry for Environment (IUF Düsseldorf, FKZ 20462296). Further, the 15‐year follow‐up examination of the LISA study was supported by the Commission of the European Communities, the 7th Framework Program: MeDALL project. This project has received funding from the European Research Council (ERC) under the European Union's Horizon 2020 research and innovation program (grant agreement No. 949906).

## CONFLICT OF INTEREST STATEMENT

The authors declare that the research was conducted in the absence of any commercial or financial relationships that could be construed as a potential conflict of interest.

## Supporting information


Appendix S1.

## Data Availability

Restrictions apply to the datasets: Due to data protection reasons, the datasets generated and/or analyzed during the current study cannot be made publicly available. The datasets are available to interested researchers from the corresponding author on reasonable request (e.g., reproducibility), provided the release is consistent with the consent given by the GINIplus and LISA study participants. Ethical approval might be obtained for the release and a data transfer agreement from the legal department of the Helmholtz Zentrum München must be accepted.
