# Supplementary material for: Symptom probability increases at low pollen exposure in adolescents with allergic rhinitis findings from the GINIplus and LISA birth cohorts
Source: Pediatr Allergy Immunol. 2026 Jul 15;37(7):e70429. doi: 10.1111/pai.70429 (PMC13373345; doi:10.1111/pai.70429)
Supplement: Supplementary file 1 — Appendix S1. [file PAI-37-e70429-s001.docx]

## **Supplementary material**

### **1 Supplementary Data**

#### **1.1 Radio-Allergo-Sorbent-test**

Radio-Allergo-Sorbent Tests (RAST) are used for the detection of specific immunoglobulin E antibodies against allergens. The CAP RAST Fluorescence Enzyme Immunoassay method (FEIA) used in this study is an in-vitro test system for the detection of IgE antibodies in the blood. The system is based on the ImmunoCAP Technology and the allergen to be tested, which is coupled with the ImmunoCAP system, reacts with the specific IgE antibodies in the patient's blood sample. Non-specific IgE antibodies are washed out and antibodies against the specific IgE are added. Unbound anti-IgE enzymes are washed out and the remaining complex of antibodies and specific IgE antibodies are incubated with a developing agent, after which the fluorescence of the eluate is measured and compared with fluorescence standards. Positive test results against the tested allergen are obtained at a cut-off of ≥ 0.35 kU/L, as used in this analysis.^1-3^

### **1.2 Identification of covariates and potential confounders**

A directed acyclic graph was constructed using the browser-based tool Dagitty to identify variables for adjustment and potential confounders.^4^ Consequently, medication use was not included in the analysis to prevent potential collider bias. Results of covariates where visualized using the forestplot package in RStudio (Figure S6).^5^

### **1.3 Pollen season definitions according to the European Academy of Allergy and Clinical Immunology**

The start of the birch pollen season was determined as the first day in any five-day span within a seven-day interval where daily counts reached at least 10 grains/m³ and totaled 100 grains/m³ or more. The end of the season was defined as the last day of the final five-day span. Grass, mugwort, and ragweed seasons were identified using the same criteria but with a daily threshold of 3 grains/m³ and a cumulative total of at least 30 grains/m³.^6^

### **1.4 Post-hoc generalized linear mixed effect models**

Post hoc generalized linear mixed models (GLMMs) were used to validate the course of the associations found. Within these models, segmented regressions before and after pollen exposure cut-offs were conducted. The cut-offs were based on changes in the slopes of the smooth terms of the GAMM models and were estimated by ascertaining the intersection points of the first derivative of the GAMM smooth terms with zero.

### **1.5 Sensitivity analysis**

*1.5.1 Methods of the sensitivity analysis*

Sensitivity analysis entailed the stratification of the dataset into GINIplus and LISA participants to test robustness between the two birth cohorts. Stratification of participants according to the date of questionnaire completion (summer or winter) was performed to determine a potential recall bias in symptom occurrence. Medication use was excluded from the adjustment variable set in the main statistical analysis to avoid a potential collider bias but was incorporated as an interaction term in the sensitivity analysis to verify the robustness of the findings.

### *1.5.2 Results of the sensitivity analysis*

Stratification of the study participants into GINI and LISA revealed a comparable course of the GAMM curve in both study groups as the findings in Figure 2A (Figure S7). This observation pertained to both the total pollen levels and lower pollen levels of less than 35 grains/m³. However, greater uncertainties were observed at elevated pollen concentrations, attributable to the reduced number of data points resulting from the division of the two groups. Furthermore, a potential variation in the responses to the questionnaire between summer and winter periods did not demonstrate a statistically significant difference in the GAMM curves (Figure S8) . Participants using medication demonstrated a higher probability of symptoms compared to non-users (Figure S9) . However, the general pattern of the GAMM smooth curves for increasing pollen exposure remained consistent with the results of the main analyses.

### **1.6 Sex-specific differences**

No significant difference in the association of pollen exposure and nasal symptoms was found between male and female adolescents ( Figure S5). This finding is consistent with the results of the study by Luyten et al.^7^ A systematic review by Pinart et al.^8^ observed that symptoms of allergic rhinitis were more prevalent in female adolescents, presumably due to genetic distinctions in allergen sensitivity next to immunological differences, symptom perception and lifestyle factors. However, our study was unable to provide evidence for a sex-specific difference in pollen susceptibility. Extant research concerning sex differences in symptoms of allergic rhinitis in adolescents, and potential for increased susceptibility in adolescent females, remains limited. Further research is therefore required to investigate possible differences in adolescents.

### **1.7 Ragweed and mugwort pollen**

No ragweed or mugwort seasons were identified in this analysis and mean monthly concentrations did not exceed 3 grains/m^3^. Consequently, our study was unable to demonstrate an association between ragweed or mugwort pollen and nasal symptoms. Previous studies conducted on ragweed pollen reported non-linear^9^ as well as linear associations^10^ between pollen exposure and nasal symptoms. Studies on associations between mugwort and allergic rhinitis are limited, with two studies from China reporting significant associations between mugwort pollen and allergic symptoms.^11,12^ Ragweed pollen are characterized by high allergenicity due to elevated pollen dispersal mechanisms and are closely related to mugwort with mutual cross-reactions.^10^ Therefore, symptoms of allergic rhinitis may be observed at low concentrations of ragweed and mugwort pollen, even in the absence of a season onset. In view of the predicted increase in ragweed pollen concentrations in Germany due to climate change^13^, an investigation of the association between ragweed pollen and allergic symptoms in Germany is recommended in future studies.

**References (Supplementary data)**

1. Kilanowski A, Chen J, Everson T, Thiering E, Wilson R, Gladish N, et al. Methylation risk scores for childhood aeroallergen sensitization: Results from the LISA birth cohort. Allergy. 2022 Sep;77(9):2803–17.

2. Blanca M, Mayorga C, Torres MJ, Reche M, Moya C, Rodriguez JL, et al. Clinical evaluation of Pharmacia CAP System^TM^ RAST FEIA amoxicilloyl and benzylpenicilloyl in patients with penicillin allergy. Allergy. 2001 Sep;56(9):862–70.

3. Mazur G, Pethran A. Detection of specific IgE in isocyanate and phthalic anhydride exposed workers: comparison of RAST RIA, Immuno CAP System FEIA, and Magic Lite SQ. Allergy. 1993 Nov;48(8):627–30.

4. Textor J, van der Zander B, Gilthorpe MS, Liśkiewicz M, Ellison GT. Robust causal inference using directed acyclic graphs: the R package ‘dagitty.’ Int J Epidemiol. 2016 Dec 1;45(6):1887–94.

5. Gordon M, Lumley T. _forestplot: Advanced Forest Plot Using “grid” Graphics_ [software]. Version 3.1.6. 2024 Nov. https://github.com/gforge/forestplot. Accessed December 12, 2024

6. Pfaar O, Bastl K, Berger U, Buters J, Calderon MA, Clot B, et al. Defining pollen exposure times for clinical trials of allergen immunotherapy for pollen‐induced rhinoconjunctivitis – an EAACI position paper. Allergy. 2017 May;72(5):713–22.

7. Luyten A, Bürgler A, Glick S, Kwiatkowski M, Gehrig R, Beigi M, et al. Ambient pollen exposure and pollen allergy symptom severity in the EPOCHAL study. Allergy. 2024 Jul;79(7):1908–20.

8. Pinart M, Keller T, Reich A, Fröhlich M, Cabieses B, Hohmann C, et al. Sex-Related Allergic Rhinitis Prevalence Switch from Childhood to Adulthood: A Systematic Review and Meta-Analysis. Int Arch Allergy Immunol. 2017;172(4):224–35.

9. Jones NR, Agnew M, Banic I, Grossi CM, Colón-González FJ, Plavec D, et al. Ragweed pollen and allergic symptoms in children: Results from a three-year longitudinal study. Sci Total Environ. 2019 Sep;683:240–8.

10. Buters J, Alberternst B, Nawrath S, Wimmer M, Traidl-Hoffmann C, Starfinger U, et al. Ambrosia artemisiifolia (Traubenkraut) in Deutschland – aktuelles Vorkommen, allergologische Bedeutung und Maßnahmen zur Eingrenzung. Allergo J. 2015 Jun;24(4):18–30.

11. Zhang J, Yan Y, Jiang F, Chen J, Ouyang Y, Zhang L. Main Airborne Pollen Species and Characteristics of Allergic Rhinitis Patients with Pollen-Related Allergies in 13 Northern Chinese Cities. J Asthma Allergy. 2024 Aug;Volume 17:757–68.

12. Lou H, Ma S, Zhao Y, Cao F, He F, Liu Z, et al. Sensitization patterns and minimum screening panels for aeroallergens in self-reported allergic rhinitis in China. Sci Rep. 2017 Aug 24;7(1):9286.

13. Bergmann KC, Brehler R, Endler C, Höflich C, Kespohl S, Plaza M, et al. Auswirkungen des Klimawandels auf allergische Erkrankungen in Deutschland. 2023 https://edoc.rki.de/handle/176904/11265. Accessed August 22, 2024

### **2 Supplementary Figures and Tables**

#### **2.1 List of Supplementary Figures**

[**Figure S1.** Study flow diagram of eligible and included participants in the data analysis and reasons for exclusion](#_Toc200551150) 6

[**Figure S2.** GAMM curves of the predicted probabilities of nasal symptom occurrence for elevating mean individual pollen exposure levels in the total study period and all participants. The solid lines represent the model estimates, and the shaded areas represent the 95% confidence intervals. The horizontal line at x = 24 indicates the plateau value. Colored rug lines along the x-axis illustrate the distribution of data points. Predictors were held constant at a mean age of 14.52 years, for family atopy in mother and father, male, non-passive smoking, no comorbidity, 0-2h time spent outside, GINI Intervention group, sensitized to house dust mites and non-sensitized to cat dander..](#_Toc200551151) 7

[**Figure S3.** GAMM curves of the predicted probability of nasal symptom occurrence for elevating mean individual pollen exposure levels during (A) birch seasons and (B) grass seasons. The solid lines represent the model estimates, and the shaded areas represent the 95% confidence intervals. The horizontal lines indicate the threshold level of the plateau effect. Colored rug lines along the x-axis illustrate the distribution of data points. Predictors were held constant at a mean age of 14.52 years, for family atopy in mother and father, male, non-passive smoking, no comorbidity, 0-2h time spent outside, GINI Intervention group, sensitized to house dust mites and non-sensitized to cat dander.](#_Toc200551152) 8

[**Figure S4**. GAMM curves of the predicted probability of nasal symptom occurrence for elevating mean individual pollen exposure levels during (A) early vs. late grass seasons and (B) grass seasons in participants sensitized to birch and grass vs. grass alone. The solid lines represent the model estimates, and the shaded areas represent the 95% confidence intervals. Colored rug lines along the x-axis illustrate the distribution of data points. Predictors were held constant at a mean age of 14.52 years, for family atopy in mother and father, male, non-passive smoking, no comorbidity, 0-2h time spent outside, GINI Intervention group, sensitized to house dust mites and non-sensitized to cat dander.](#_Toc200551153) 9

[**Figure S5.** GAMM curves of the partial association of mean individual pollen exposure on probability of nasal symptom occurrence in the total study period and male and female participants. The solid line represent the model estimates, and the shaded areas represent the 95% confidence intervals. The horizontal line at x = 24 indicates the plateau value. Colored rug lines along the axes illustrate the distribution of data points and n represents the number of person-months included in the model. Models were adjusted for age, sex, family atopy, passive smoking, asthma or eczema, time spent outside and sensitization to house dust mites or cat dander.](#_Toc200551154) 10

**Figure S6.** Odds ratios, 95% confidence intervals and p-values for predictors included in the model. The forest plot was created using the forestplot package in RStudio. * Indicates p-values less than or equal to 0.05………………………………………………11 Figure S7. GAMM curves of the partial association of mean individual pollen exposure on probability of nasal symptom occurrence during (A) the whole study period and (B) for lower pollen exposures in GINIplus and LISA cohorts. The solid lines represent the model estimate, and the shaded areas represent the 95% confidence intervals. Colored rug lines along the x-axis illustrate the distribution of data points. Models were adjusted for age, sex, family atopy, passive smoking, asthma or eczema, time spent outside and sensitization to house dust mites or cat dander…………………………………………………………………………..……………..12 Figure S8. GAMM curves of the partial association of mean individual pollen exposure on probability of nasal symptom occurrence during (A) the whole study period and (B) for lower pollen exposures for questionnaire completion in summer vs. winter. The solid lines represent the model estimate, and the shaded areas represent the 95% confidence intervals. Colored rug lines along the x-axis illustrate the distribution of data points. Models were adjusted for age, sex, family atopy, passive smoking, asthma or eczema, time spent outside and sensitization to house dust mites or cat dander………………………………………………………..……….13 Figure S9. GAMM curves of the partial association of mean individual pollen exposure on probability of nasal symptom occurrence during (A) the whole study period and (B) for lower pollen exposures after including medication use. The solid lines represent the model estimate and the shaded areas represent the 95% confidence intervals. Colored rug lines along the x-axis illustrate the distribution of data points. Models were adjusted for age, sex, family atopy, passive smoking, asthma or eczema, time spent outside and sensitization to house dust mites or cat dander…………………………………………………………………………………………14

**LISA:**

3097 participants enrolled at birth

**GINIplus:**

5991 participants enrolled at birth

**GINIplus:**

3198 participants at 15-year questionnaire

**LISA:**

1740 participants at 15-year questionnaire

**GINIplus + LISA:** 1474 participants

**Exclusion:** No answers provided for symptoms: 58

**GINIplus + LISA:** 1532 participants

**GINIplus Munich:**

1594 participants at 15-year questionnaire

**LISA Munich:**

930 participants at 15-year questionnaire

**Exclusion:** Not participated in parent questionnaire: 3

**Exclusion:** Not participated in parent questionnaire: 6

**GINIplus + LISA:** 2515 participants

**Exclusion:** Not participated in sensitization tests: 983

**GINIplus + LISA:** 17688 person-months included in the analysis

Symptom-reporting for previous **12** months per participant

**Figure S1.** Study flow diagram of eligible and included participants in the data analysis and reasons for exclusion


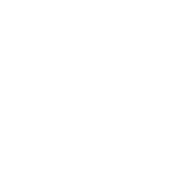


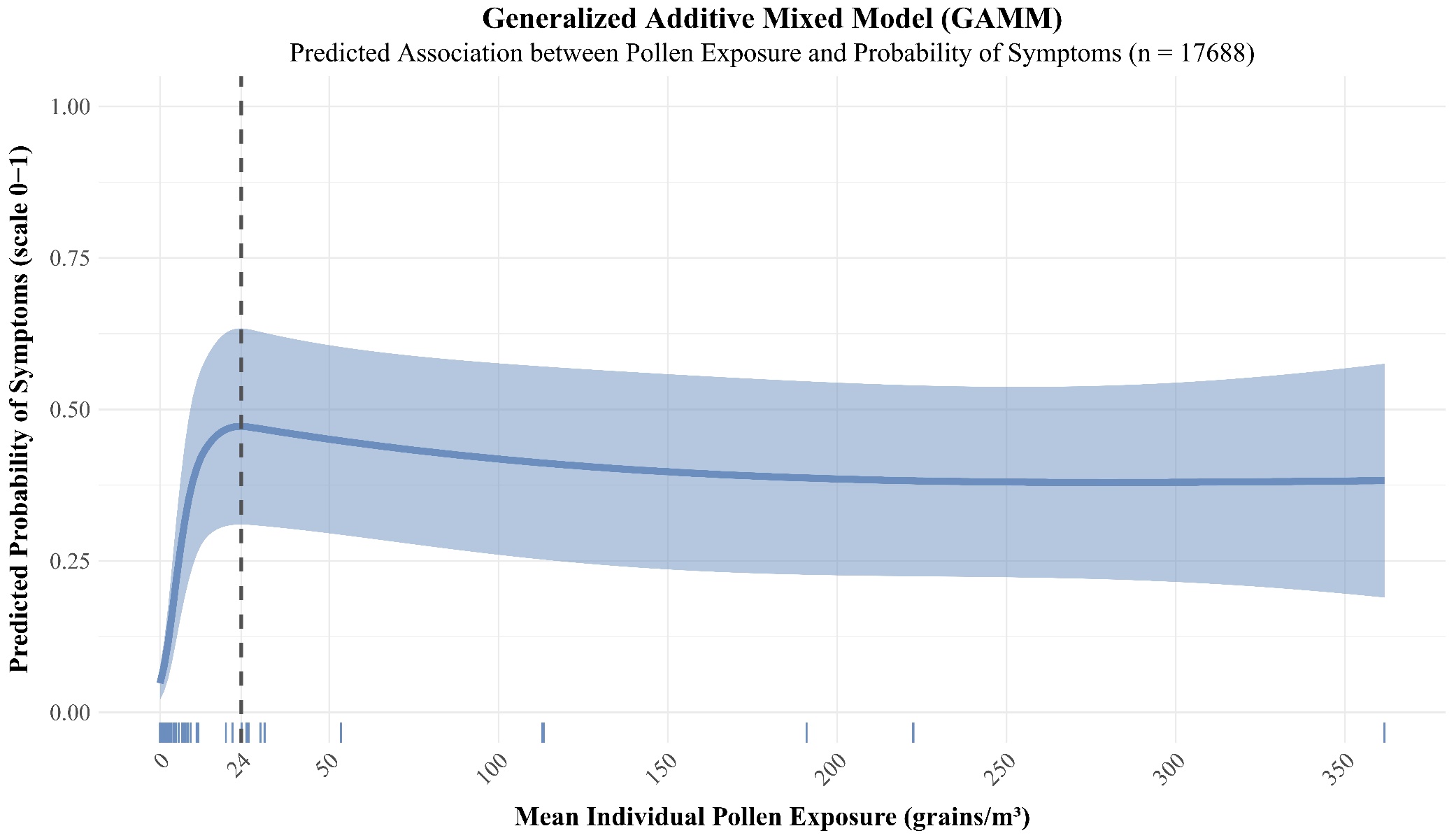


**Mean Individual Pollen Exposure (grains/m^3^)**

**Mean Individual Pollen Exposure (grains/m^3^)**

**Probability of symptom occurrence (0-1)**

**Probability of symptom occurrence (0-1)**

**Predicted probabilities of symptoms for pollen exposure** GAMM term including all covariates in the prediction (n= 17688)

**Figure S2.** GAMM curves of the predicted probabilities of nasal symptom occurrence for elevating mean individual pollen exposure levels in the total study period and all participants. The solid lines represent the model estimates, and the shaded areas represent the 95% confidence intervals. The horizontal line at x = 24 indicates the plateau value. Colored rug lines along the x-axis illustrate the distribution of data points. Predictors were held constant at a mean age of 14.52 years, for family atopy in mother and father, male, non-passive smoking, no comorbidity, 0-2h time spent outside, GINI Intervention group, sensitized to house dust mites and non-sensitized to cat dander.

(A)

(A)

**Mean Individual Birch Pollen Exposure (grains/m^3^)**

**Mean Individual Birch Pollen Exposure (grains/m^3^)**

**Probability of symptom occurrence (0-1)**

**Probability of symptom occurrence (0-1)**

**Predicted probabilities of symptoms for pollen exposure during Birch Seasons** GAMM term including all covariates in the prediction (n= 2901)

**Predicted probabilities of symptoms for pollen exposure during Birch Seasons** GAMM term including all covariates in the prediction (n= 2901)


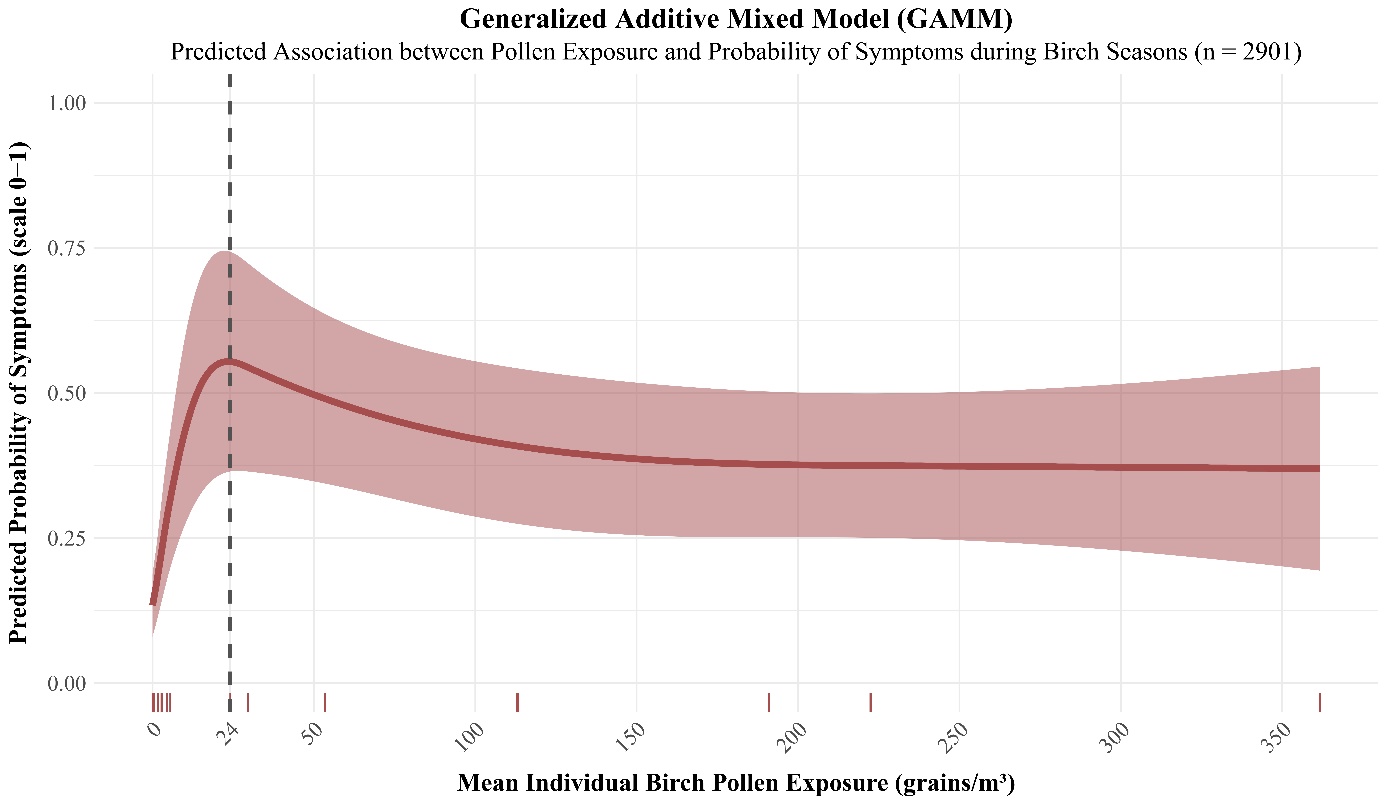


(B)

(B)

**Mean Individual Grass Pollen Exposure (grains/m^3^)**

**Mean Individual Grass Pollen Exposure (grains/m^3^)**

**Probability of symptom occurrence (0-1)**

**Probability of symptom occurrence (0-1)**

**Predicted probabilities of symptoms for pollen exposure during Grass Seasons** GAMM term including all covariates in the prediction (n= 5524)

**Figure S3.** **Predicted probabilities of symptoms for pollen exposure during Grass Seasons** GAMM term including all covariates in the prediction (n= 5524)


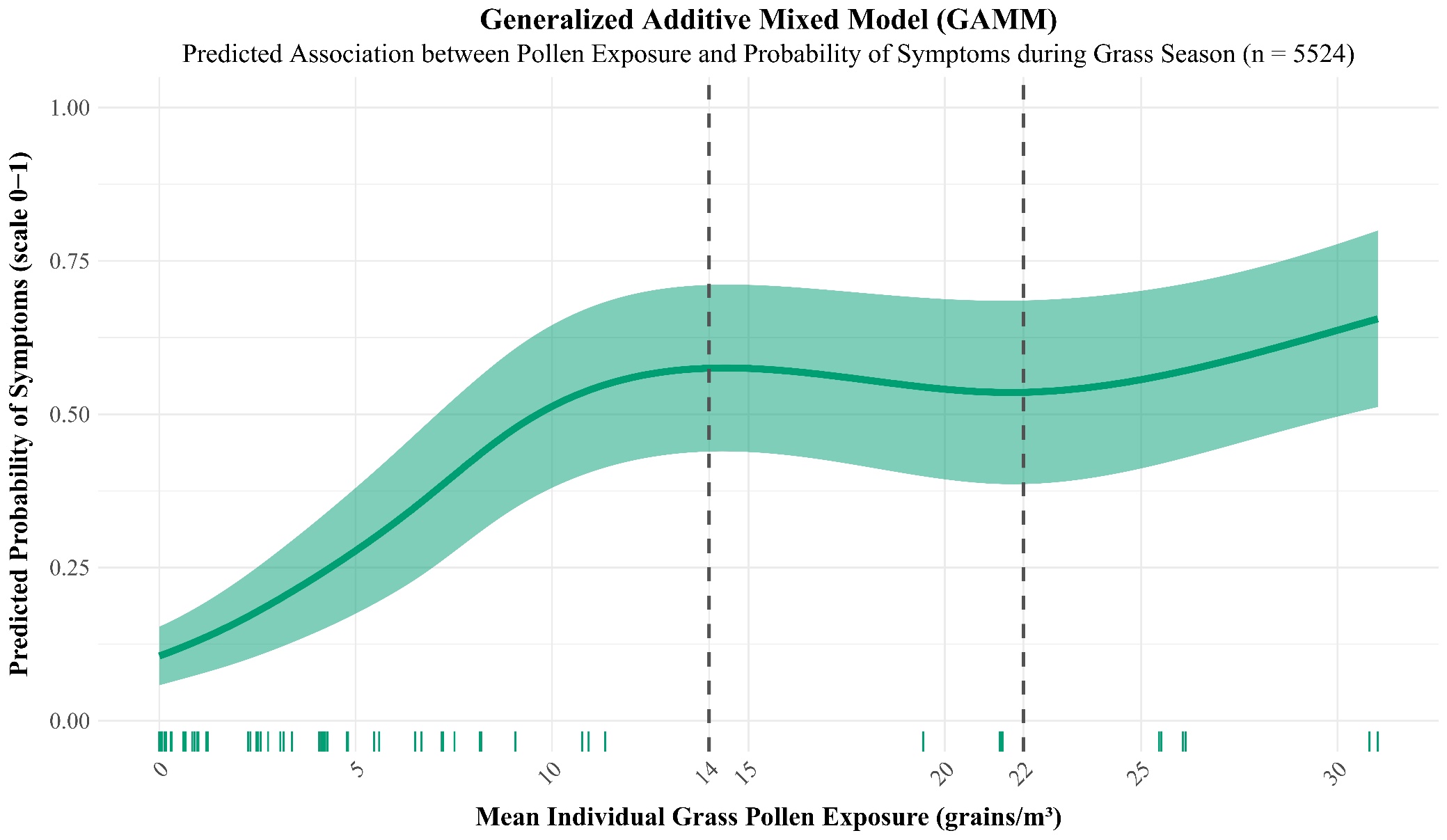


**Figure S3.** GAMM curves of the predicted probability of nasal symptom occurrence for elevating mean individual pollen exposure levels during (A) birch seasons and (B) grass seasons. The solid lines represent the model estimates, and the shaded areas represent the 95% confidence intervals. The horizontal lines indicate the threshold level of the plateau effect. Colored rug lines along the x-axis illustrate the distribution of data points. Predictors were held constant at a mean age of 14.52 years, for family atopy in mother and father, male, non-passive smoking, no comorbidity, 0-2h time spent outside, GINI Intervention group, sensitized to house dust mites and non-sensitized to cat dander.

**Figure S3.** GAMM curves of the predicted probability of nasal symptom occurrence for elevating mean individual pollen exposure levels during (A) birch seasons and (B) grass seasons. The solid lines represent the model estimates, and the shaded areas represent the 95% confidence intervals. The horizontal lines indicate the threshold level of the plateau effect. Colored rug lines along the x-axis illustrate the distribution of data points. Predictors were held constant at a mean age of 14.52 years, for family atopy in mother and father, male, non-passive smoking, no comorbidity, 0-2h time spent outside, GINI Intervention group, sensitized to house dust mites and non-sensitized to cat dander.

**Mean Individual Grass Pollen Exposure (grains/m^3^)**

**Mean Individual Grass Pollen Exposure (grains/m^3^)**

**Probability of symptom occurrence (0-1)**

**Probability of symptom occurrence (0-1)**

**Predicted probabilities of symptoms for pollen exposure during early vs. late grass seasons** GAMM term including all covariates in the prediction (early n= 2423 / late n=3101)


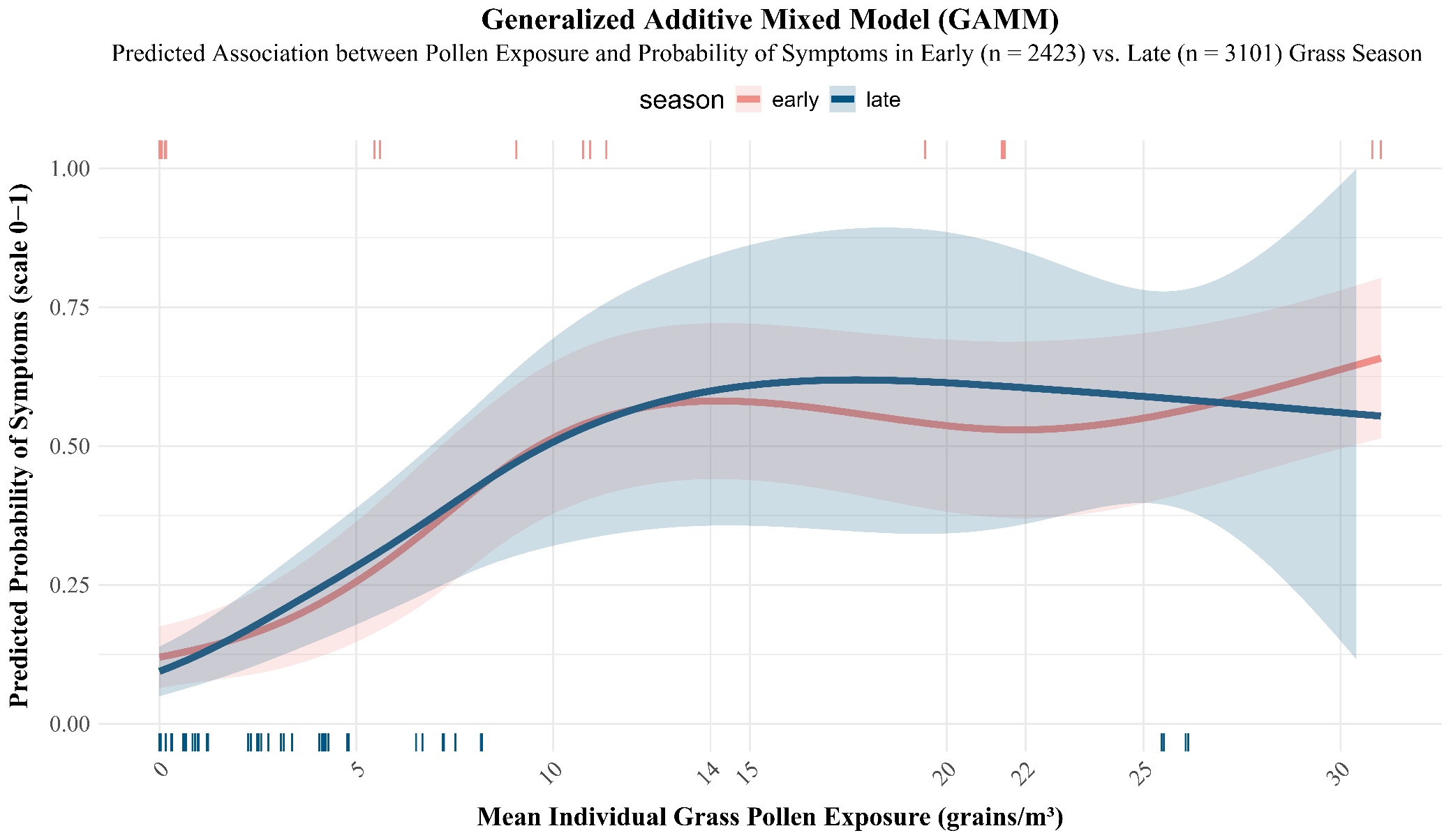


**Mean Individual Grass Pollen Exposure (grains/m^3^)**

**Mean Individual Grass Pollen Exposure (grains/m^3^)**

**Probability of symptom occurrence (0-1)**

**Probability of symptom occurrence (0-1)**

**Predicted probabilities of symptoms for pollen exposure (sensitized to birch & grass vs. grass)** GAMM term including all covariates in the prediction (birch & grass n= 1290 / grass n=688)


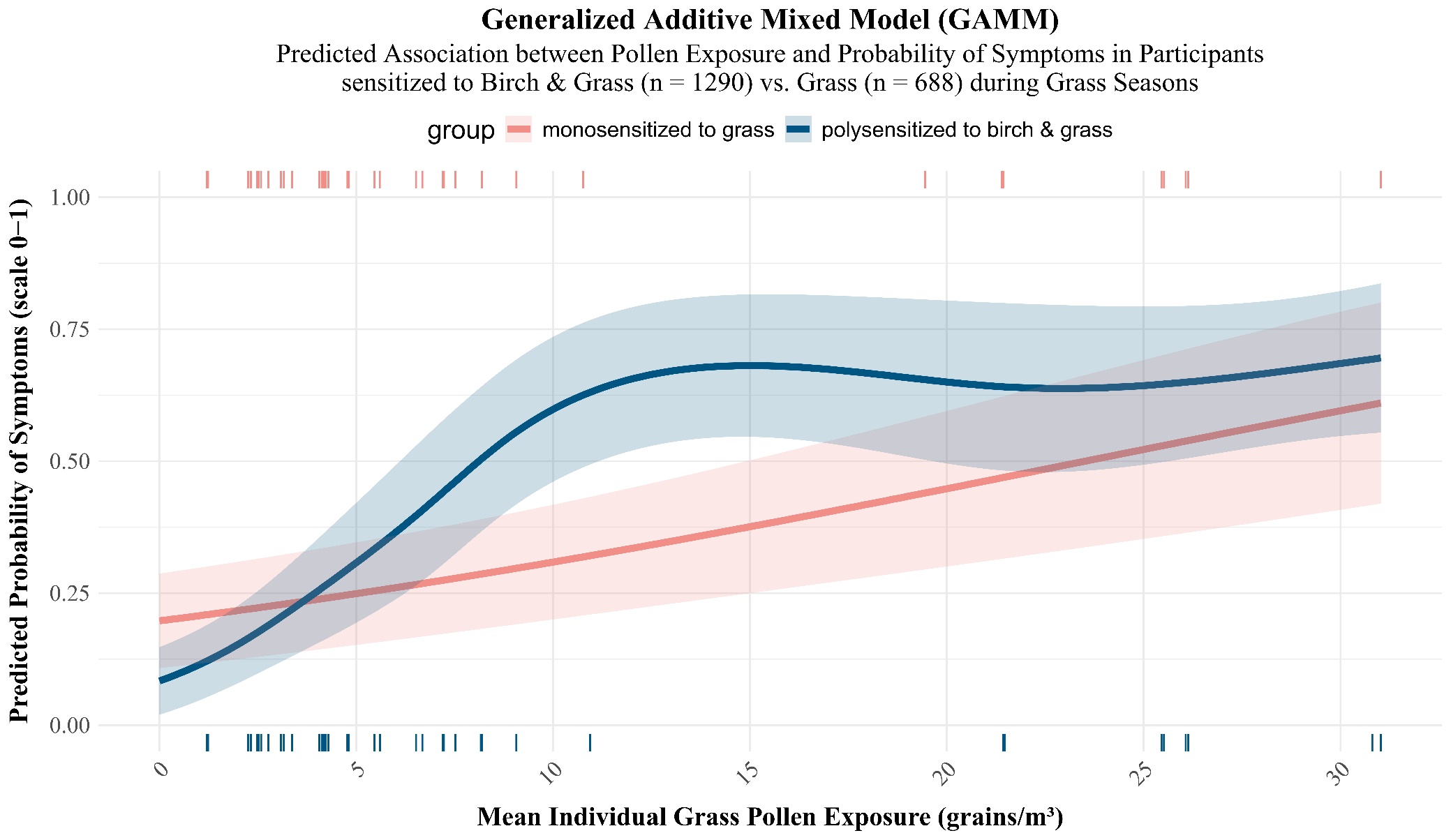


(A)

(A)

(B)

(B)

**Figure S4**. GAMM curves of the predicted probability of nasal symptom occurrence for elevating mean individual pollen exposure levels during (A) early vs. late grass seasons and (B) grass seasons in participants sensitized to birch and grass vs. grass alone. The solid lines represent the model estimates, and the shaded areas represent the 95% confidence intervals. Colored rug lines along the x-axis illustrate the distribution of data points. Predictors were held constant at a mean age of 14.52 years, for family atopy in mother and father, male, non-passive smoking, no comorbidity, 0-2h time spent outside, GINI Intervention group, sensitized to house dust mites and non-sensitized to cat dander.


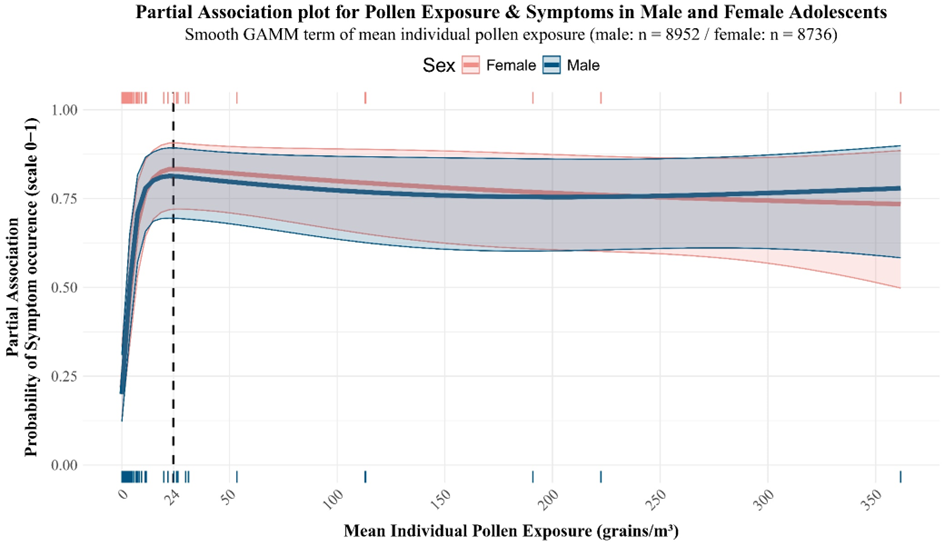


**Mean Individual Pollen Exposure (grains/m^3^)**

**GAMM term of mean individual pollen exposure (male n= 8952 / female n= 8736)** Association plot for pollen exposure & symptoms in male & female adolescents

**Probability of symptom occurrence (0-1)**

**Figure S5.** GAMM curves of the partial association of mean individual pollen exposure on probability of nasal symptom occurrence in the total study period and male and female participants. The solid line represents the model estimates, and the shaded areas represent the 95% confidence intervals. The horizontal line at x = 24 indicates the plateau value. Colored rug lines along the axes illustrate the distribution of data points and n represents the number of person-months included in the model. Models were adjusted for age, sex, family atopy, passive smoking, asthma or eczema, time spent outside and sensitization to house dust mites or cat dander.


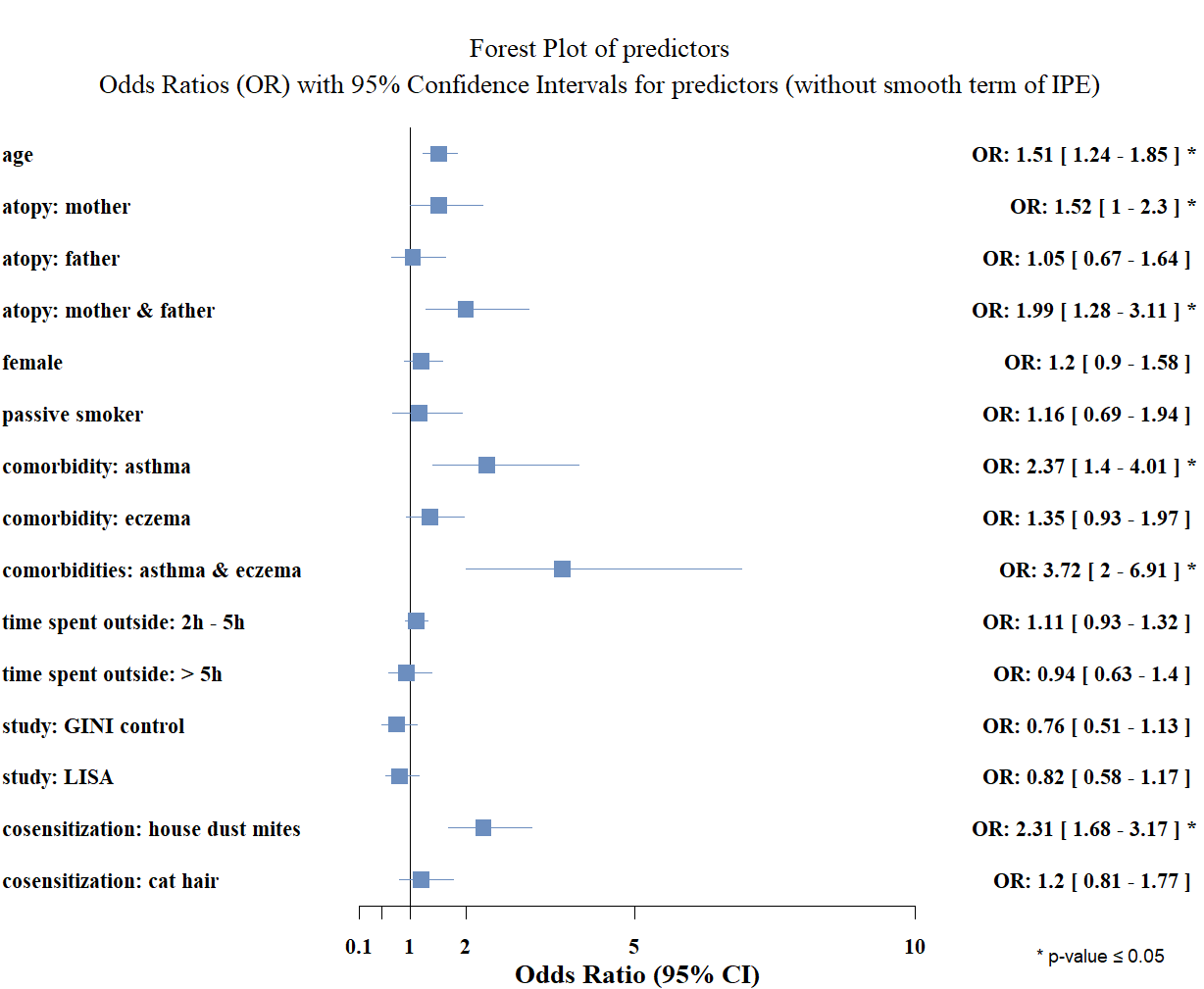


**Figure S6.** Odds ratios, 95% confidence intervals and p-values for predictors included in the model. The forest plot was created using the forestplot package in RStudio. * Indicates p-values less than or equal to 0.05

(A)

**GAMM term of mean individual pollen exposure (GINIplus n= 11700 / LISA n= 5988)** Association plot for pollen exposure & symptoms in GINIplus & LISA participants


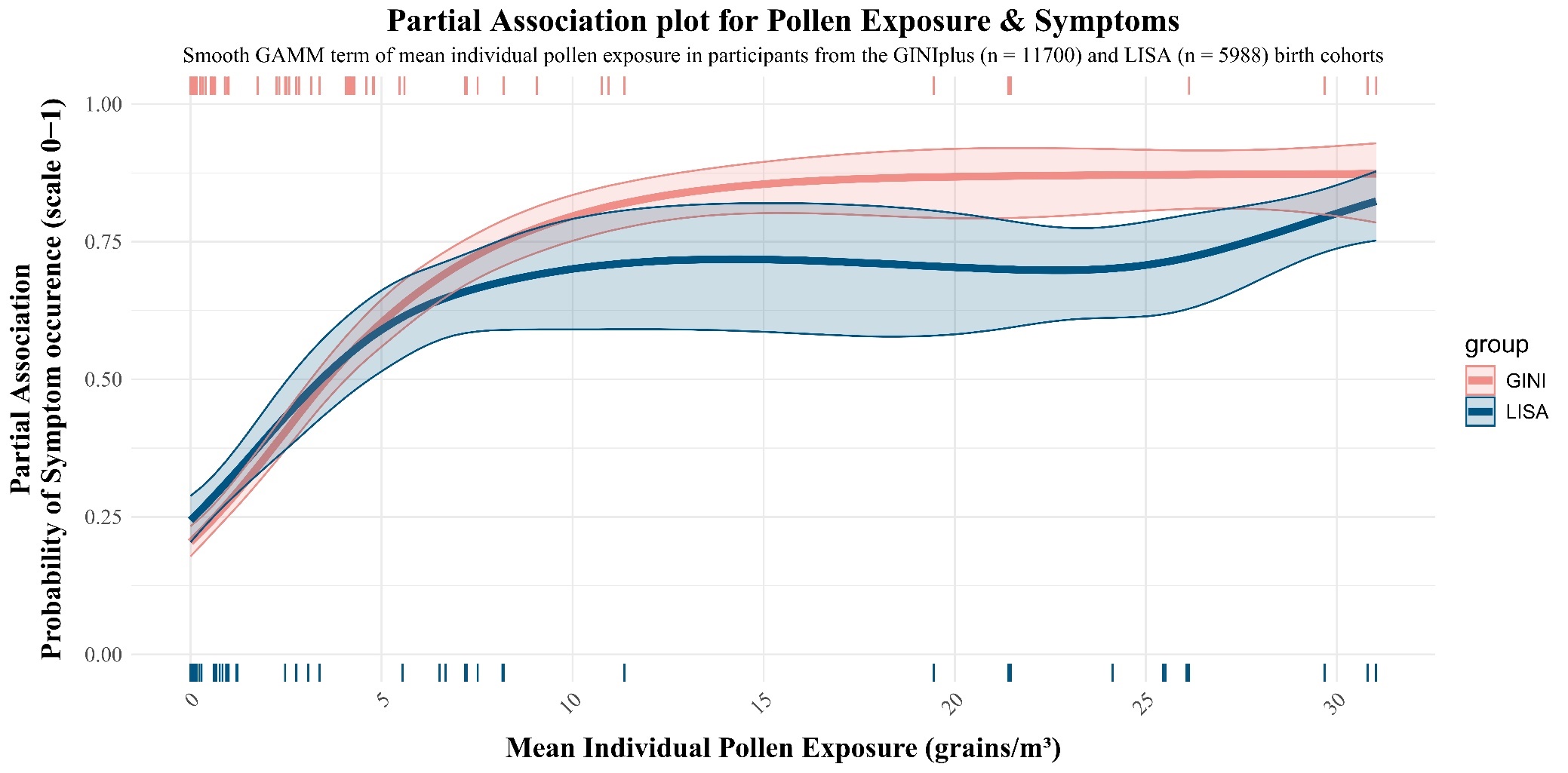

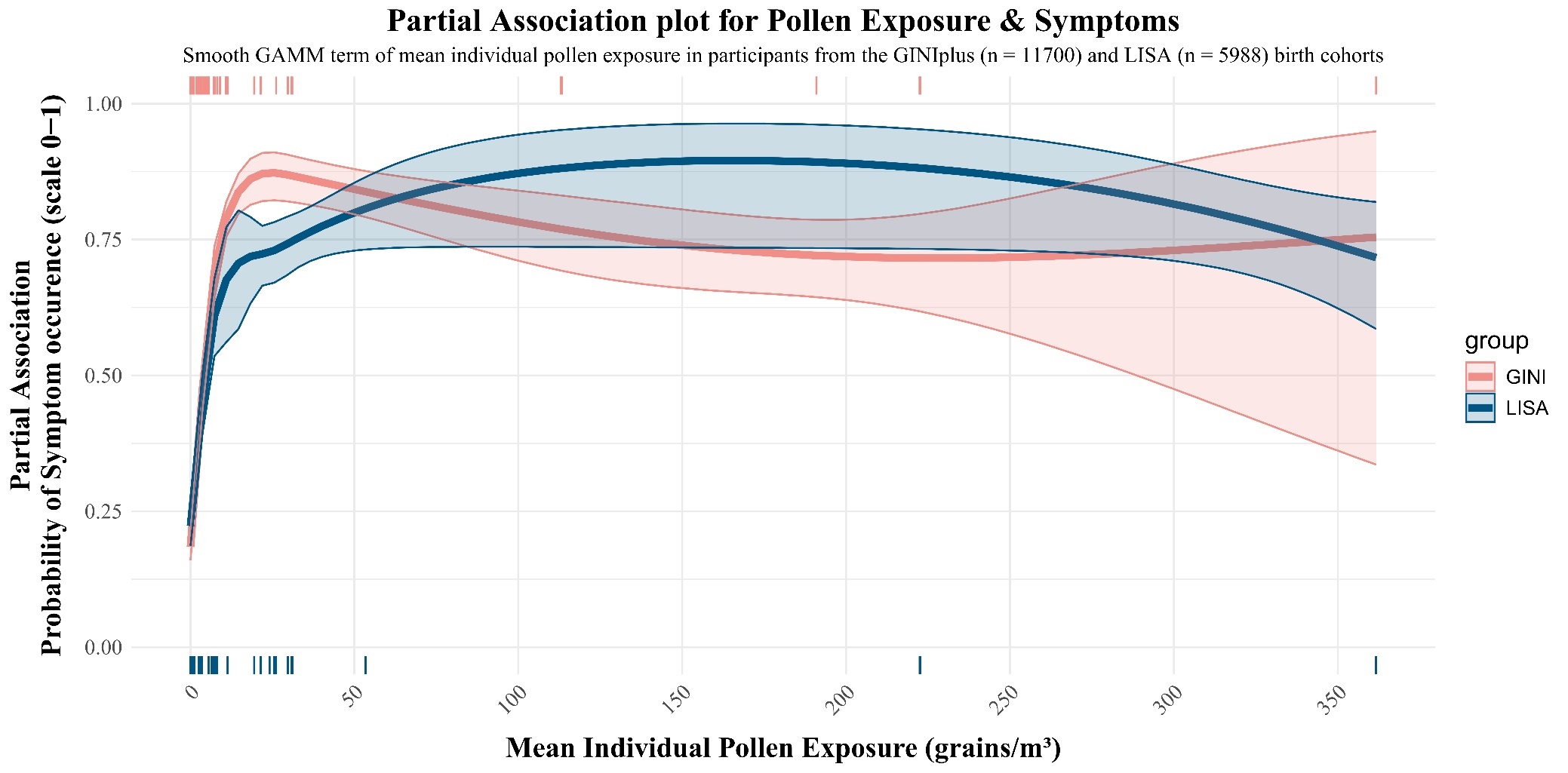

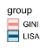

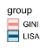


(B)

**Mean Individual Pollen Exposure (grains/m^3^)**

**Figure S7.** GAMM curves of the partial association of mean individual pollen exposure on probability of nasal symptom occurrence during (A) the whole study period and (B) for lower pollen exposures in GINIplus and LISA cohorts. The solid lines represent the model estimate, and the shaded areas represent the 95% confidence intervals. Colored rug lines along the x-axis illustrate the distribution of data points. Models were adjusted for age, sex, family atopy, passive smoking, asthma or eczema, time spent outside and sensitization to house dust mites or cat dander.

**GAMM term of mean individual pollen exposure (GINIplus n= 11700 / LISA n= 5988)** Association plot for pollen exposure & symptoms in GINIplus & LISA participants

**Probability of symptom occurrence (0-1)**

**Mean Individual Pollen Exposure (grains/m^3^)**

**Probability of symptom occurrence (0-1)**

GINI LISA

GINI LISA

GINI LISA


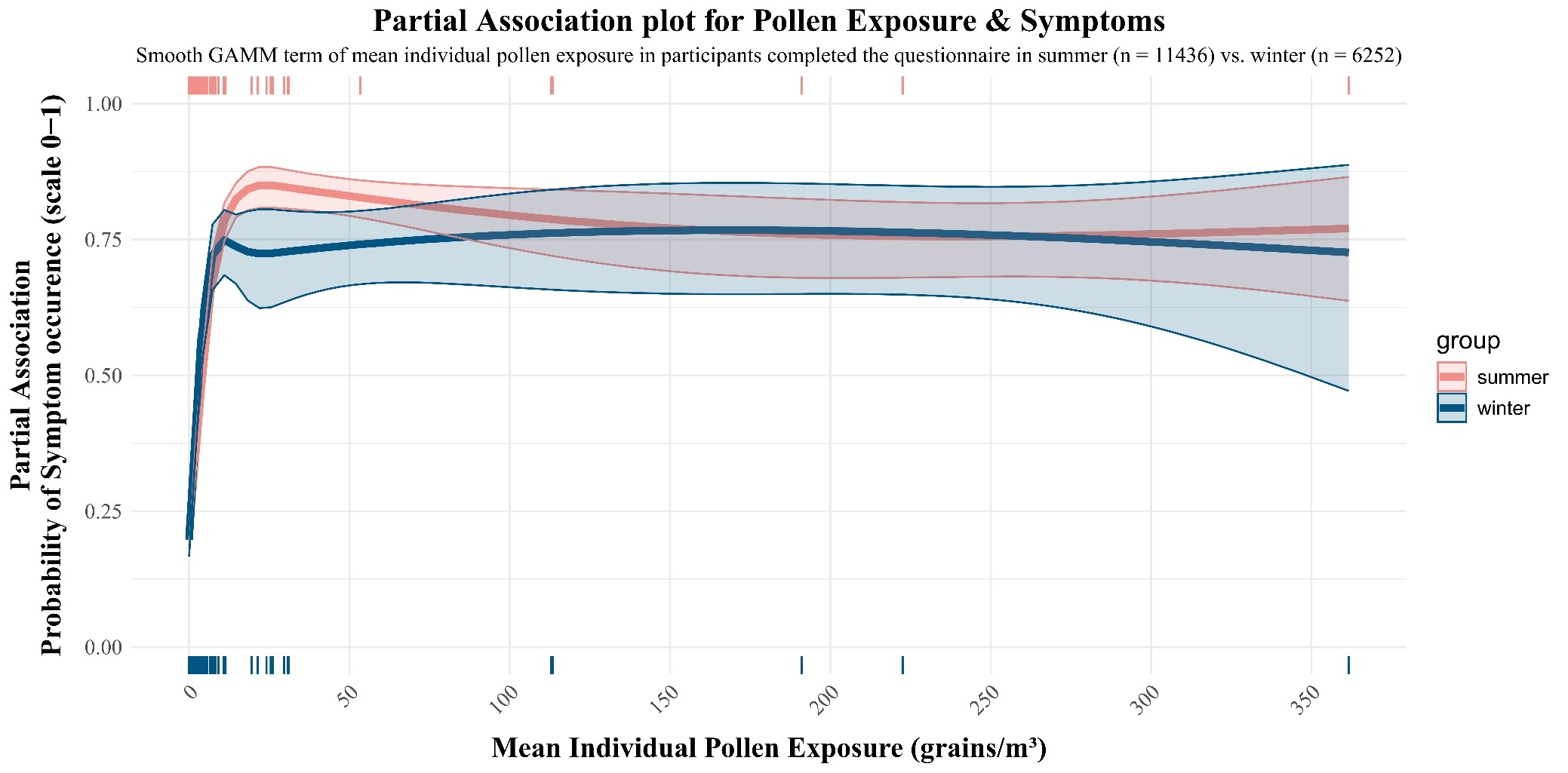

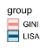


(A)

**GAMM term of mean individual pollen exposure (summer n= 11436 / winter n= 6252)** Association plot for pollen exposure & symptoms in summer & winter

**Mean Individual Pollen Exposure (grains/m^3^)**

**Probability of symptom occurrence (0-1)**

summer winter


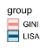

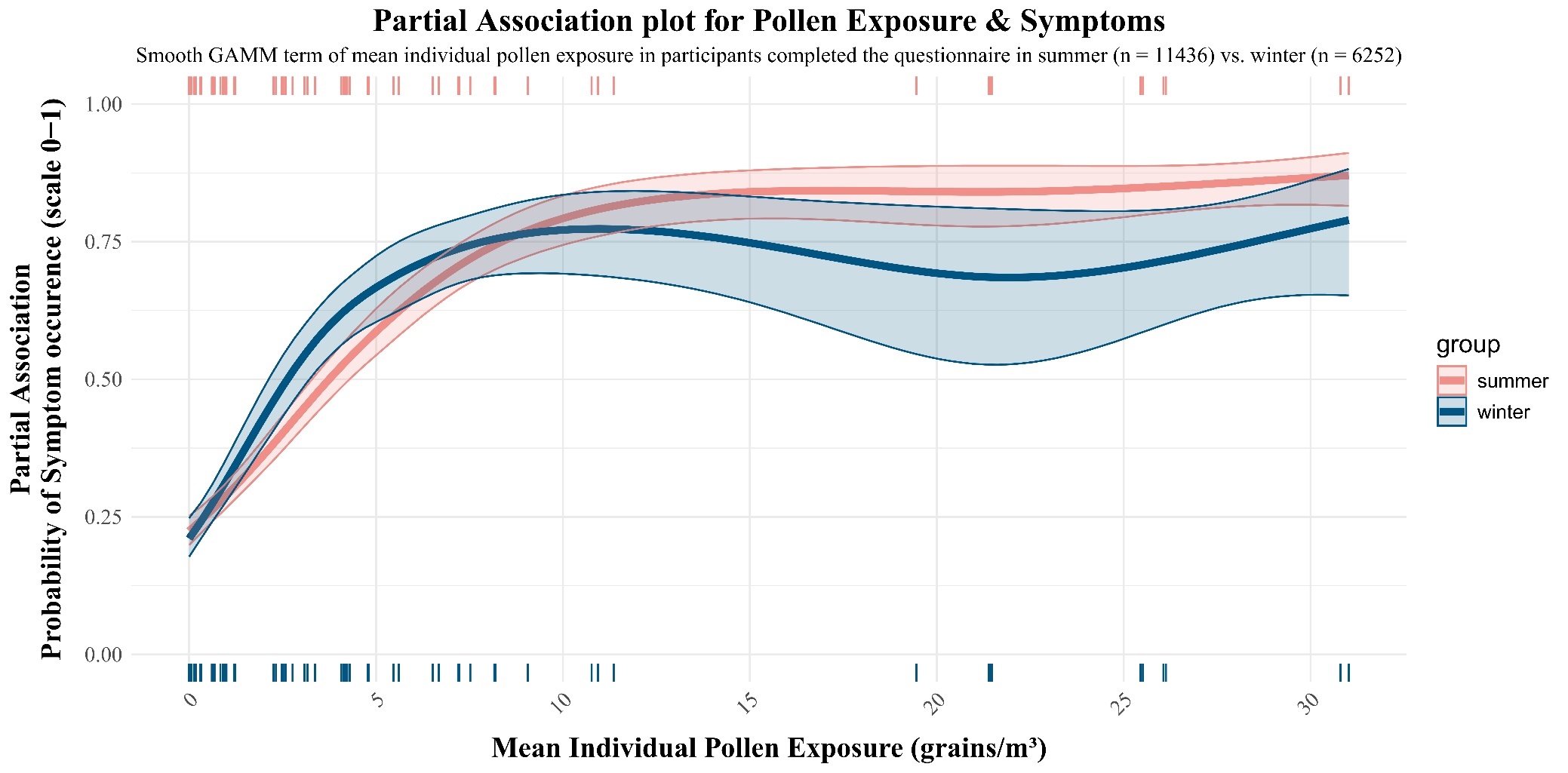


**Figure S8.** GAMM curves of the partial association of mean individual pollen exposure on probability of nasal symptom occurrence during (A) the whole study period and (B) for lower pollen exposures for questionnaire completion in summer vs. winter. The solid lines represent the model estimate, and the shaded areas represent the 95% confidence intervals. Colored rug lines along the x-axis illustrate the distribution of data points. Models were adjusted for age, sex, family atopy, passive smoking, asthma or eczema, time spent outside and sensitization to house dust mites or cat dander.

(B)

**GAMM term of mean individual pollen exposure (summer n= 11436 / winter n= 6252)** Association plot for pollen exposure & symptoms in summer & winter

**Probability of symptom occurrence (0-1)**

summer winter

**Mean Individual Pollen Exposure (grains/m^3^)**


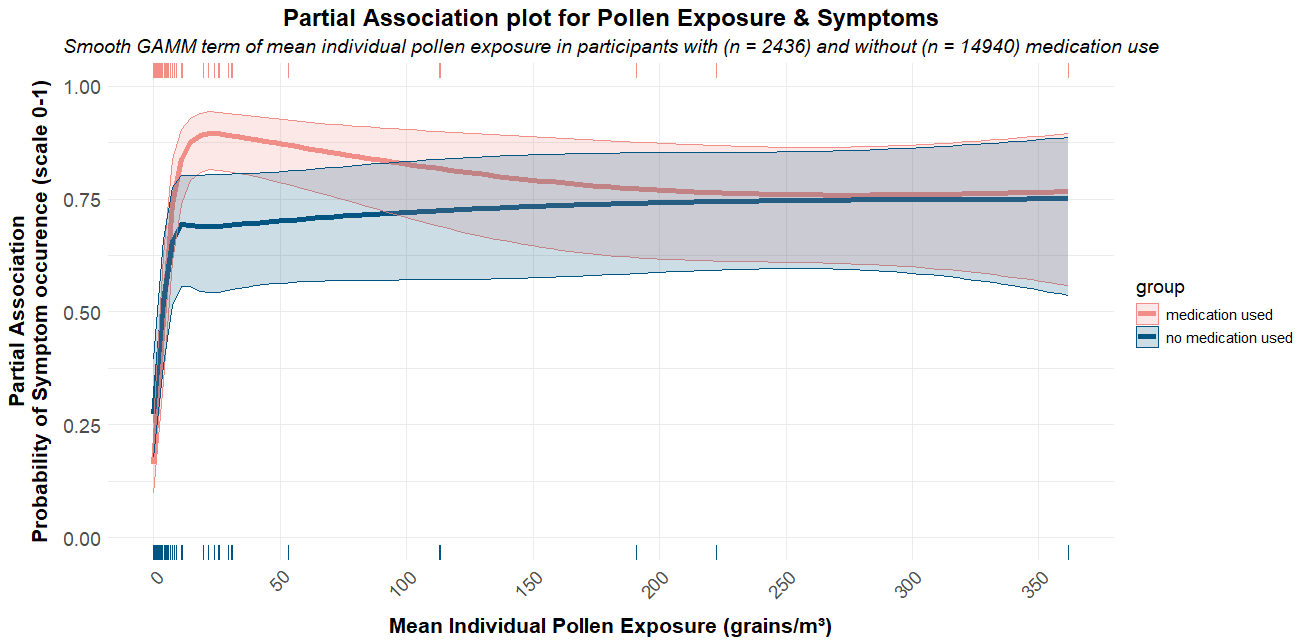

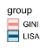

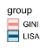


(A)

**GAMM term of mean individual pollen exposure (med n= 2436 / no med n= 14940)** Association plot for pollen exposure & symptoms with vs. without medication use

**Mean Individual Pollen Exposure (grains/m^3^)**

medication use no medication use

**Figure S9.** GAMM curves of the partial association of mean individual pollen exposure on probability of nasal symptom occurrence during (A) the whole study period and (B) for lower pollen exposures after including medication use. The solid lines represent the model estimate and the shaded areas represent the 95% confidence intervals. Colored rug lines along the x-axis illustrate the distribution of data points. Models were adjusted for age, sex, family atopy, passive smoking, asthma or eczema, time spent outside and sensitization to house dust mites or cat dander.

**Probability of symptom occurrence (0-1)**

(B)

**GAMM term of mean individual pollen exposure (med n= 2436 / no med n= 14940)** Association plot for pollen exposure & symptoms with vs. without medication use


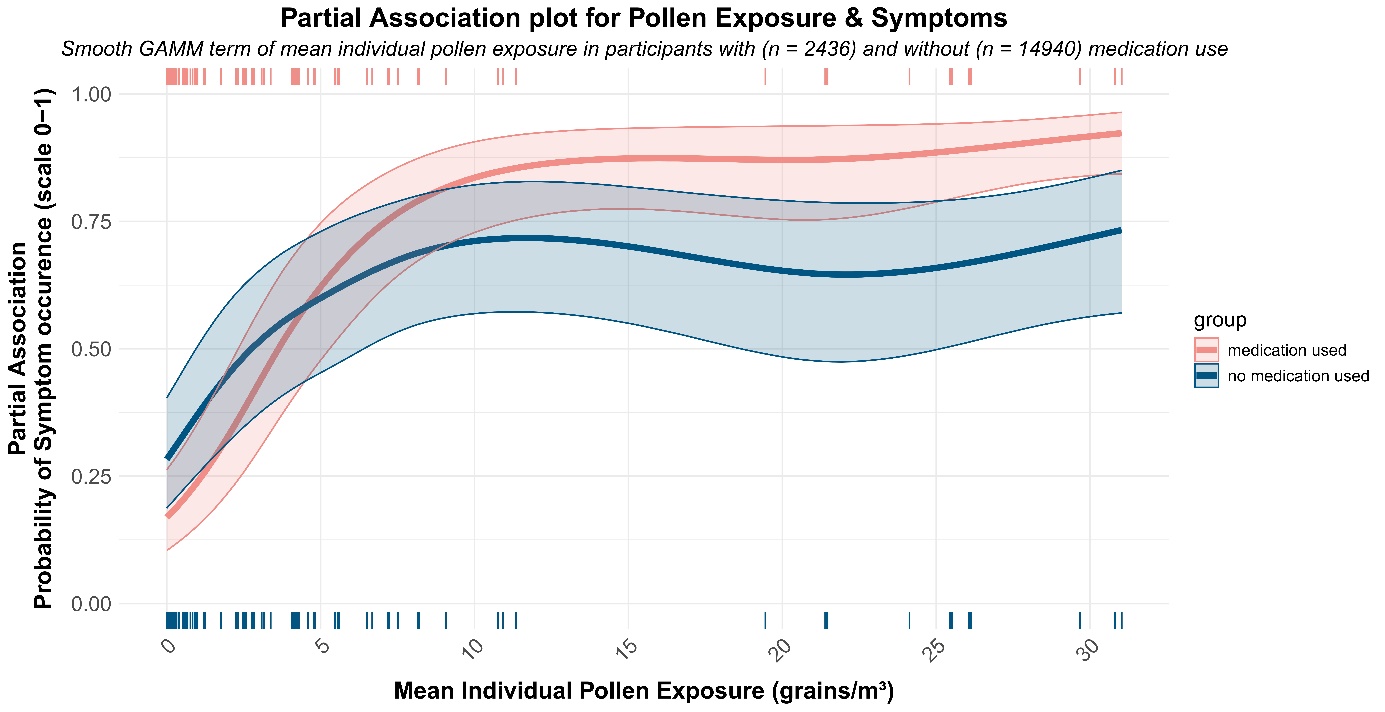


medication use no medication use

**Probability of symptom occurrence (0-1)**

**Mean Individual Pollen Exposure (grains/m^3^)**
